# Deploying synthetic coevolution and machine learning to engineer protein-protein interactions

**DOI:** 10.1126/science.adh1720

**Published:** 2023-07-28

**Authors:** Aerin Yang, Kevin M. Jude, Ben Lai, Mason Minot, Anna M. Kocyla, Caleb R. Glassman, Daisuke Nishimiya, Yoon Seok Kim, Sai T. Reddy, Aly A. Khan, K. Christopher Garcia

**Affiliations:** 1 Department of Molecular and Cellular Physiology, Stanford University School of Medicine, Stanford, CA 94305, USA.; 2 Howard Hughes Medical Institute, Stanford University School of Medicine, Stanford, CA 94305, USA.; 3 Toyota Technological Institute at Chicago, Chicago, IL 60637, USA; 4 Department of Biosystems Science and Engineering, ETH Zurich, Basel, Switzerland.; 5 Departments of Pathology, and Family Medicine, University of Chicago, Chicago, IL 60637, USA; 6 Department of Structural Biology, Stanford University School of Medicine, Stanford, CA 94305, USA.

## Abstract

Fine-tuning of protein-protein interactions occurs naturally through coevolution, but this process is difficult to recapitulate in the laboratory. We describe a synthetic platform for protein-protein coevolution that can isolate matched pairs of interacting muteins from complex libraries. This large dataset of coevolved complexes drove a systems-level analysis of molecular recognition between Z domain-affibody pairs spanning a wide range of structures, affinities, cross-reactivities, and orthogonalities, and captured a broad spectrum of coevolutionary networks. Furthermore, we harnessed pre-trained protein language models to expand, *in silico*, the amino acid diversity of our coevolution screen, predicting remodeled interfaces beyond the reach of the experimental library. The integration of these approaches provides a means of generating protein complexes with diverse molecular recognition properties as tools for biotechnology and synthetic biology.

## One-Sentence Summary

Integration of synthetic coevolution with machine learning enables prediction of remodeled protein-protein interfaces.

In evolutionary biology, the concept of coevolution underscores the compensatory relationships between biological systems that occur as a result of evolutionary pressures. Coevolution refers to reciprocal changes that occur under selective pressures between pairs of biomolecules or living organisms to fine-tune functions. Charles Darwin introduced the concept of coevolution by observing the relationship between the length of insects’ proboscis and the size of orchids’ spur, which led him to predict the evolutionary changes of insects that could suck from the deep spur of Darwin’s orchid (1). By analogy, interacting proteins often undergo coupled mutations within, or proximal to their interfaces to maintain or refine their functional interactions (2-4). Phylogenetic sequence information reveals correlated mutations accumulate through natural evolution, suggestive of compensatory changes occurring between interacting residues (5, 6).

Protein coevolution has been difficult to study experimentally in the laboratory using reconstituted systems. Although directed evolution via phage or yeast surface display has enabled efficient screening to discover binders with improved affinity and specificity toward a fixed target protein (7), it has been more challenging to execute “library-on-library” selections to coevolve both sides of a protein-protein interface concurrently (8-10). *In vitro*, co-selection by mixing separate libraries is limited by the inability to isolate discrete coevolved pairs from complex mixtures, thereby losing connectivity between the sequences of members of interacting pairs (8). Coevolution studies using both *in vivo* functional selections such as bacterial *in vivo* screening (11, 12) or yeast two-hybrid systems (13) and *in vitro* screening strategies including yeast mating systems (9, 10) or compartmentalized two-hybrid system (14) have been reported, but these systems are limited by small library sizes resulting in acquisition of sparse information rather than a broad evolutionary spectrum.

An additional practical limitation to developing a synthetic coevolution system is that the diversity of experimental combinatorial libraries is limited, which makes experimental exploration of the entire sequence space required to fully sample a protein-protein interface impossible. However, recent advances in protein language models (15, 16), and transfer learning offer the possibility of employing transfer learning to “transfer” knowledge learned from a subset of combinations to predict the binding affinity of a larger set of amino acid combinations that have not been experimentally tested. This enables effective exploration of a much larger space of combinations and identification of those that perform the desired function,

A high-throughput system for coevolving protein-protein interfaces could have practical utility for protein engineering in biotechnology and serve as a powerful basic tool to interrogate fundamental properties of molecular recognition. Here, we describe a strategy to achieve coevolution of protein-protein pairs using a high-throughput screening platform for library-on-library based directed evolution. We adopted the Z domain of staphylococcal protein A and its affibody binder dimer complex as a model system (17). The large dataset of interacting mutant sequences was subjected to systems-level analysis of molecular recognition. High resolution crystal structures of orthogonal mutant pairs elaborated compensatory changes in predicted co-varying residues and structural adaptations. By tracking the mutational trajectories of coevolved mutants, we observed continuous changes in the connectivity and specificity between mutants. We show that the set of coevolved protein pairs can inform machine learning algorithms to predict new complexes with amino acid compositions not encoded within the experimental libraries.

## Results

### Design of inter-protein coevolution and validation of selection strategy

**Figure 1.**
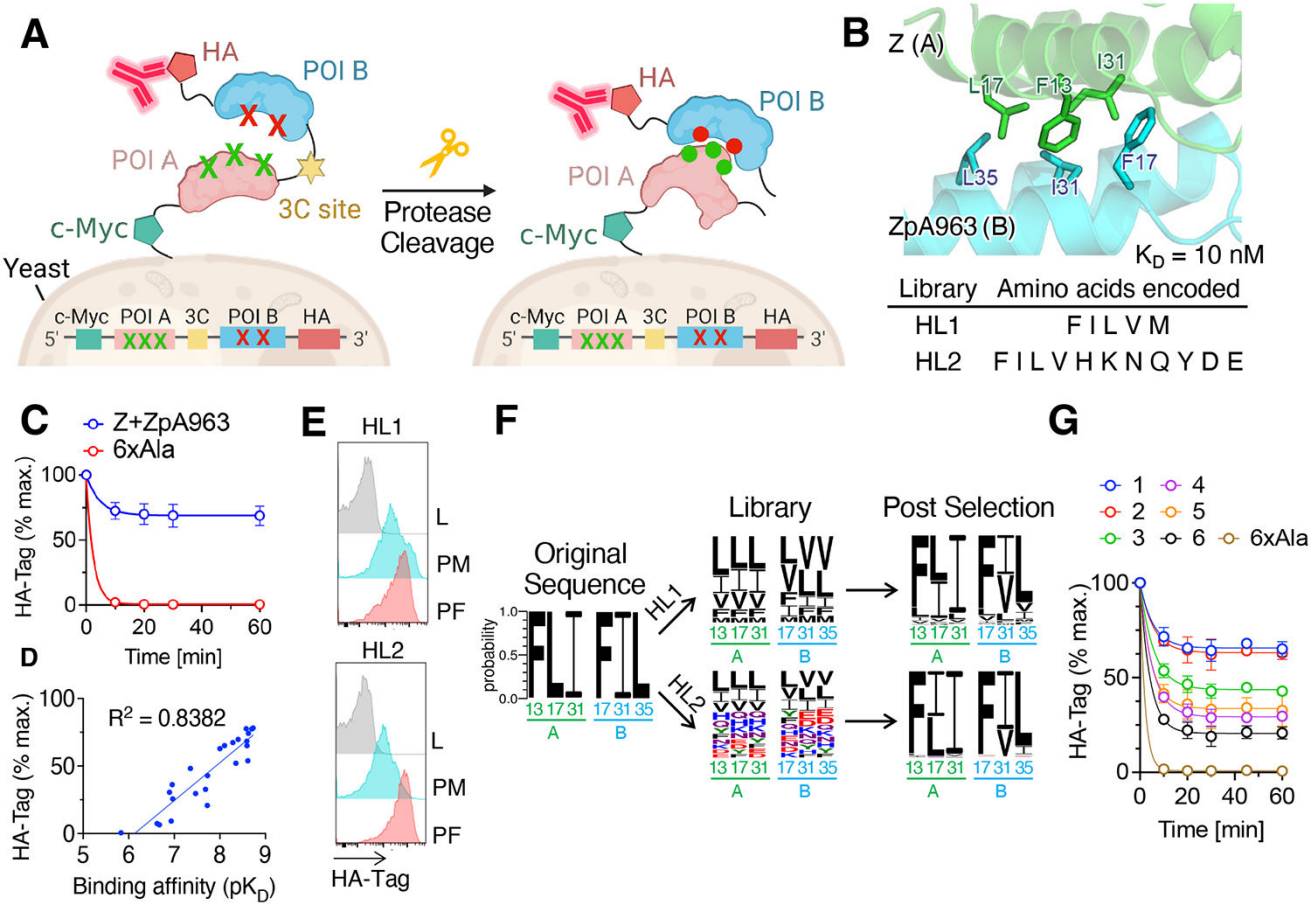
Design and validation of protein-protein coevolution strategy. (A) A schematic representation of protein-protein coevolution workflow. The α-agglutinin yeast surface display system was used to display two proteins connected by a flexible linker. A 3C protease site within the linker enabled cleavage, and the interacting proteins can be captured by C-terminally bound anti-HA antibody (red). (B) Close-up view of key residues in the hydrophobic cavity of Z domain (green) and affibody ZpA963 (blue) (PDB: 2M5A). Encoded amino acids are used for two separate libraries, HL1 and HL2 (bottom). (C) On-yeast cleavage-capture assay of interacting pair (Z+ZpA963) and non-interacting pair (6xAla). Data are mean ± SD; n = 3 independent replicates. (D) Correlation between on-yeast cleavage-capture assay and binding affinity measured of Z domain-affibody dimer mutants measured by SPR. Note that on-yeast cleavage-capture assay shows a strong semilog-linear relationship (R^2^ = 0.8382) with binding affinity (pK_D_). (E) Histogram of the flow cytometric analysis. Note that HA-tag fluorescence in the library shows strong enrichment after MACS (PM) and FACS (PF) for HL1 and HL2 libraries. (F) Sequence frequency logo of NGS data in the naïve library and post final round of FACS. The original sequence (FLI+FIL) is derived from Z domain (A) and ZpA963 (B) dimer. Note that the libraries converged back to the original sequences either exactly or with minimal variations. The color scheme represents hydrophobic (black), polar (green), basic (blue), acidic (red), and neutral (purple) amino acids. (G) On-yeast cleavage-capture assay of the six most frequent mutants from HL1 and HL2 NGS data. The sequence of each mutant (1: FII+FIL, 2: FLI+FIL, 3: FII+FVL, 4: FLI+FVL, 5: FLI+FII, 6: FII+FII) Note that all six mutants show different levels of steady-state binding of HA-tag fluorescence during 3C protease cleavage. Data are mean ± SD; n = 3 independent replicates.

To develop a platform for protein-protein coevolution using yeast surface display, we adapted the yeast display α-agglutinin system to display two different proteins expressed as a single chain connected by a flexible linker (Fig. 1A). A 3C protease site (-LEVLFQGP-) was inserted within the linker to enable 3C protease cleavage of the connected proteins. Following proteolytic cleavage, the first protein and its associated c-Myc tag remain covalently attached to the yeast cell surface while the second protein and HA-tag are liberated. The surviving non-covalently connected interacting pairs, together with the associated yeast clones, can then be isolated with C-terminal HA-tag binding antibodies. The identities of both interacting proteins can then be determined by DNA sequencing of the enriched yeast clones.

We wished to execute proof-of-concept experiments for this strategy using a simple system of small stable proteins, so we chose the complex (K_D_ = 10 nM) of Z domain and its affibody binder, ZpA963 (PDB: 2M5A). This is a model system with an interface idealized through phage display (18). We tested the cleavage-capture efficiency of three forms of fluorescently labeled anti-HA tag antibodies with different valency (Fab, IgG mAb, Fab+streptavidin (SA) complex) to determine whether their fluorescence was maintained after 3C protease cleavage of the linker between two proteins (Fig. S1A). We found that both bivalent IgG mAb labeled cells and tetrameric complex (Fab+SA), but not monovalent Fab labeled cells, maintained their staining levels of 45.8% and 69.9% of uncleaved cells respectively after 3C cleavage (Fig. 1A). We then chose six key residues forming the central hydrophobic portion of the interface based on the NMR structure of dimeric Z+ZpA963, accounting for 406 Å^2^ of the 1662 Å^2^ buried solvent-accessible surface area (BSA) on the two protein chains (Fig. 1B). When these six residues (F13, L17, and I31 in Z, and F17, I31, and L35 in ZpA963) were each mutated to alanine (6xAla), the antibody-stained yeast cells quickly lost their fluorescence to 0.63% within 10 minutes after 3C protease cleavage, whereas interacting pair displaying cells still retained fluorescence to 68.7% after an hour (Fig. 1C). We optimized different linker lengths (18, 22, 26 AA) and various components (1 copy or 2 copies of 3C protease site, HA-tag in the linker or at C-terminus), and magnetic-activated cell sorting (MACS) selection in the cleavage-capture assay (Fig. S1B-S1F). These general considerations and optimization strategies can be applied to other protein-protein complexes one wishes to implement into this coevolution platform. Notably, the on-yeast cleavage-capture assay is highly correlated with the dimer binding affinity, showing a log-linear relationship (R^2^ = 0.8382) at submicromolar affinity range (Fig. 1D).

As an initial test, we asked whether the high-affinity Z-domain complex with ZpA963 would converge back to its phage-display idealized interface through coevolution (Fig. 1B). We generated libraries by randomizing the aforementioned six positions with two sets of degenerate codons: one set with only minimal hydrophobic amino acids (F, I, L, V, and M) and the other set with a more diverse set amino acids (F, I, L, V, H, K, N, Q, Y, D, and E) (Fig. 1B). After each round of selection, library evolution was monitored by cleavage-capture assay and flow cytometry. After four or five rounds of positive MACS selections along with interspersed negative selections, both HL1 and HL2 libraries clearly enriched higher HA-tag fluorescence after 30 min of 3C protease cleavage (Fig. 1E). We isolated cells displaying interacting pairs by FACS from each round of MACS for further next-generation sequencing (NGS). The NGS results showed that the libraries converged to the original sequences exactly or with very few differences (Fig. 1F). Leu17 in Z domain (A) and Ile31 in ZpA963 (B) were replaceable with Ile or Val, while other sites strongly converged to the original amino acids (Fig. 1F). Each clone was assessed by the cleavage-capture assay and reached different levels of steady-state binding of HA-tag fluorescence during 3C protease cleavage (Fig. 1G). Using surface plasmon resonance (SPR), we measured binding affinities that ranged from 7.9 nM to 34.1 nM, similar to the original template dimer affinity of 10 nM (Fig. S2A and S2B). These data suggest that the coevolution strategy was able to remodel the protein interface to its original “optimal” state from non-ideal starting points represented in the complex libraries.

### Coevolution of a low affinity dimer creates optimized new interfaces.

**Figure 2.**
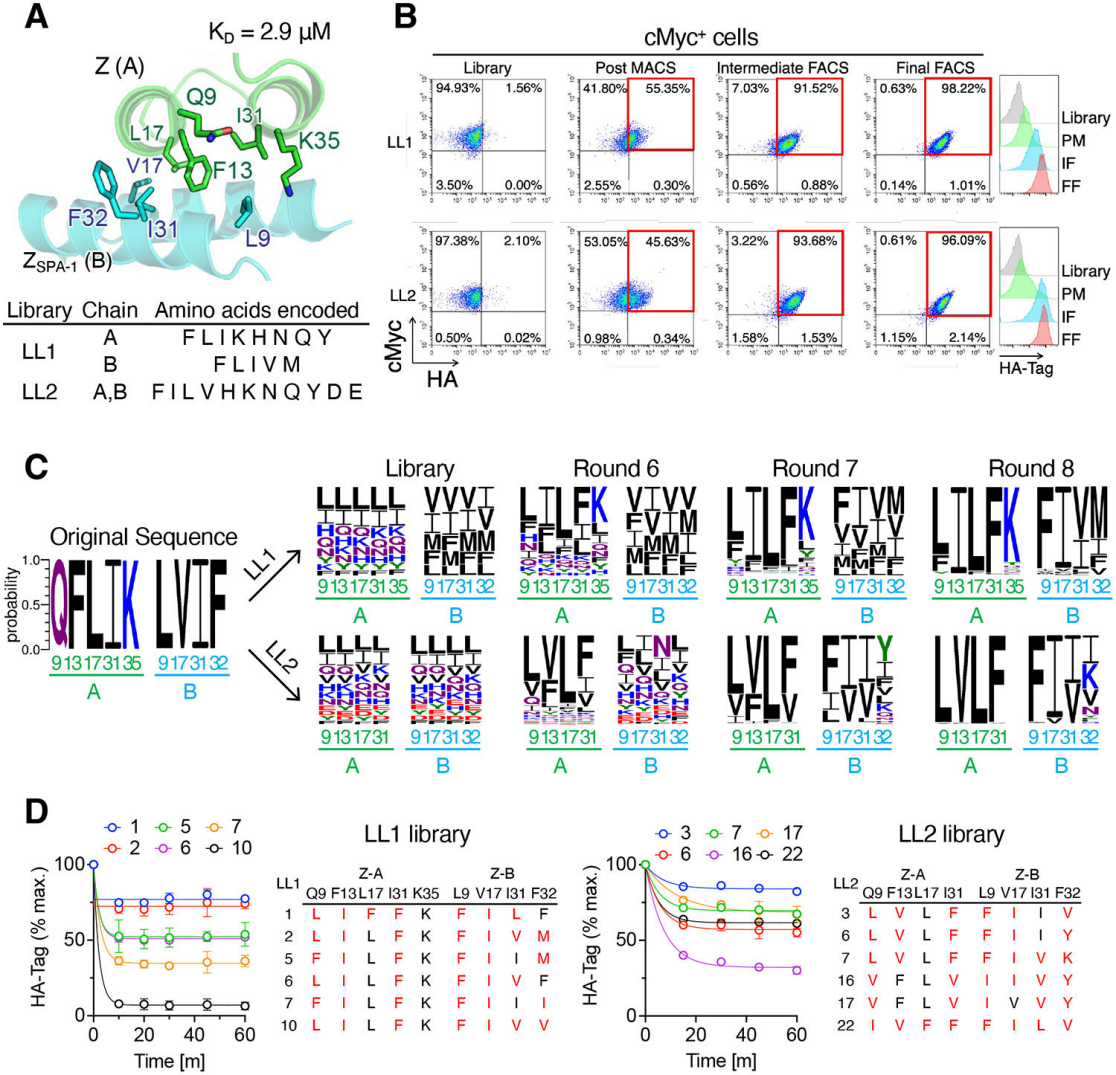
Engineering remodeled dimer interfaces by coevolution. (A) Library positions on the interface (top) from the complex of Z domain (green, chain A) and Z_SPA-1_ (blue, chain B) (PDB: 1LP1). Encoded amino acids used for making two separate libraries, LL1 and LL2 (bottom). (B) Flow cytometry dot plots showing enrichment of HA-tag fluorescence (red squares) in the library after rounds 6 to 8 (left). Antibody-labeled yeast cells were cleaved with 3C protease for 30 min. Cells were pre-gated on c-Myc+. Histograms showing elevation of HA-tag fluorescence during selection, from round 6 (green), to 7 (blue) and 8 (red) (right). (C) Sequence frequency logo of NGS data in naïve library, rounds 6, 7, and 8, revealing the appearance of consensus sequences as the selection proceeded in both LL1 and LL2 libraries. The original sequence (QFLIK+LVIF) is derived from Z domain (A) and Z_SPA-1_ (B) dimer. The color scheme represents hydrophobic (black), polar (green), basic (blue), acidic (red), and neutral (purple) amino acids. (D) On-yeast cleavage-capture assay of the mutants from LL1 (left) and LL2 (right) library. The altered positions compared to original amino acids are colored in red. Data are mean ± SD; n = 3 independent replicates.

We next generated libraries at the interface of a weakly associating dimer (Z+ Z_SPA-1_) with micromolar affinity to determine if we could affinity-mature the interface by coevolution (19, 20) (Fig. 2A). Consistent with its low affinity, the Z+ Z_SPA-1_ pair rapidly lost its HA-tag fluorescence within 15min of 3C protease treatment (Fig. S2C). Based on the crystal structure of the complex (PDB: 1LP1), nine interfacial positions located in a central hydrophobic patch were selected for library randomization: five positions (Q9, F13, L17, I31, K35) from Z domain and four positions (L9, V17, I31, F32) from Z_SPA-1_ domain. The first library, LL1, was designed to use minimal codon sets encoding both polar and hydrophobic amino acids (F, L, I, K, H, N, Q, and Y) for five positions on the Z domain and hydrophobic amino acids (F, L, I, V, and M) for four positions on the affibody Z_SPA-1_ (Fig. 2A). The second library, LL2, used a more diverse codon set encoding mixed amino acids (F, I, L, V, H, K, N, Q, Y, D, and E) for four randomized positions on each of the Z domain and the affibody, so that the functional diversity (1.91 × 10^9^) of the yeast library almost reached the theoretical nucleotide diversity (4.29 × 10^9^). After six rounds of positive MACS and two rounds of FACS selections, more than 90% of the populations enriched into the upper right quadrant of flow cytometry dot plots (Fig. 2B). NGS data collected at each step of selection clearly revealed the appearance of consensus sequences as the selection proceeded (Fig. 2C). Based on the sequencing data, we tested 11 clones from LL1 and 22 clones from LL2 using the cleavage-capture assay, and all reached varying levels of steady-state binding during 1-hour 3C protease treatment (Fig. 2D). In contrast to the result of coevolution from the high affinity pair, the enriched mutants from both LL1 and LL2 libraries have only a few conserved residues shared with the original template: on average, 6 to 7 mutations were enriched (Fig. 2C and 2D). The highest affinity mutant from each library achieved approximately three-log enhanced affinity, K_D_ of 1.99 nM for LL1.c1 (LIFFK/FILF) and 1.86 nM for LL2.c3 (LVLF/FIIV) compared to the original dimer (K_D_ = 2.92 μM) (Fig. S2D-S2G).

### Synthetic coevolution yields pairs with different specificities and cross-reactivities

**Figure 3.**
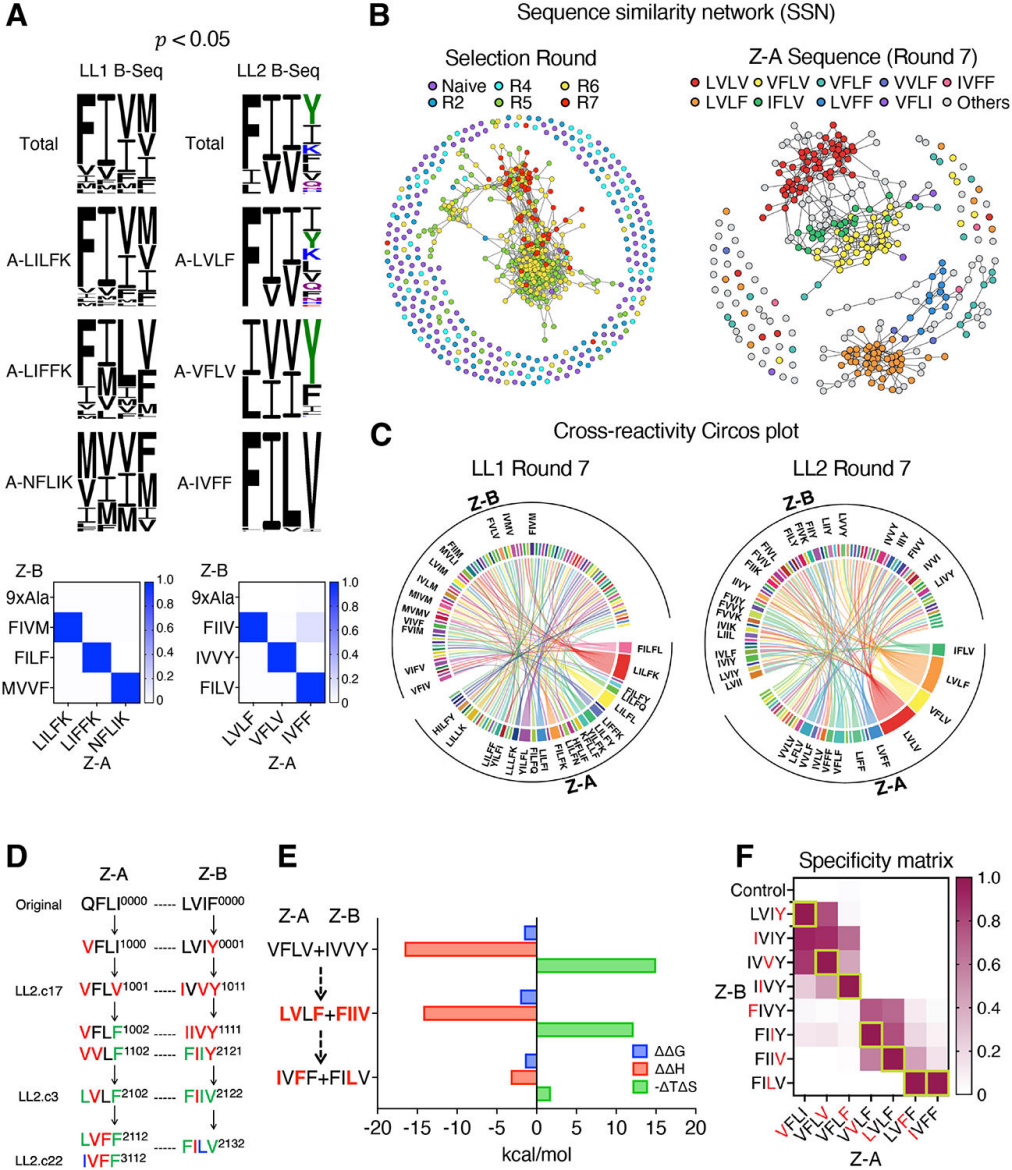
Visualization and mapping of coevolutionary networks. (A) The sequence logo of Z-B sequences paired with each Z-A sequence from the statistically enriched NGS data (p-value < 0.05) and actual binding specificity measured by on-yeast cleavage-capture assay, normalized to the highest affinity of each Z-A sequence (below). Filtered sequences accurately predicted binding specificity, matching the actual binding specificity of each Z-A sequence. (B) Sequence similarity networks (SSNs) of concatenated 8 amino acid Z-A/Z-B library position sequences from all screening rounds (left) and round 7 (right) of LL2 library. Notable Z-A sequences are colored and specified in the panel (right). The edit distance threshold for connecting nodes in the total library network is 2 and in the round 7 network is 1. The left SSN is colored by screening round and demonstrates connectivity among sequences from later screening rounds (rounds 5 to 7). The right SSN is colored by Z-A sequence and provides a detailed view of the enriched stage (round 7), showing cluster formation based on Z-A specificities. (C) Circos cross-reactivity plot of 100 sampled pairs from LL1 and LL2 round 7 sequence data. The Circos plots illustrate the pairwise relationships between the 100 sampled pairs of Z-A and Z-B proteins. Each pair is normalized to have equal area, providing a visual representation of the approximate cross-reactivity of each sequence. (D) A single mutational pathway of mutants from the LL2 library connecting the original sequence (QFLI/LVIF) with the prominent LL2 library mutants. Mutated positions are color-coded: red (one mutation), green (two mutations), and blue (three mutations). The number of mutations at each position is represented by a 4-digit number next to each Z-A and Z-B sequence (E) A plot illustrating the changes in ΔΔG, ΔΔH, and −ΔTΔS for three mutants in the pathway (D) compared to the original pair (QFLI/LVIF). Mutations introduced in each step are highlighted in red. (F) A matrix to show binding specificity changes of the Z-A variants from the pathway. Binding affinities measured by on-yeast cleavage-capture assay were normalized based on the highest affinity in each Z-A sequence. The single mutation introduced at each step is indicated in red. The highest affinity pair in each column was boxed in green. Control is a mutant with all library positions mutated to alanines. Data are mean of n = 3 independent replicates. .

To characterize the relationship between coevolved protein sequences in our screen, we visualized the sequencing data as a network. We used statistical enrichment to identify the sequence pairs with the strongest likelihood of binding from the enriched library sequencing data, based on the overall count of the individual sequences in the screen. We used a hypergeometric test (see Methods) to calculate this enrichment statistic, which compares the observed frequency of a particular protein pair in a screening library to the expected frequency of the pair based on the overall count of the individual proteins in the library. If the observed frequency is significantly higher than the expected frequency, it suggests that the protein pair is enriched for interaction. We extracted sequences with a p-value < 0.05 for further visualization and analysis of cross-reactivity and specificity. The enriched sequences accurately predicted the binding specificity of each Z-A sequence, matching well with its actual binding specificity (Fig. 3A).

The sequence similarity network (SSN) is an efficient way to observe relationships among large sets of evolutionarily related proteins (21). We constructed SSNs using the concatenated Z-A and Z-B full-length 8 amino acid sequences collected from all screening rounds of the LL2 library. The SSN revealed clear connectivity between sequences from later rounds (rounds 5 to 7) when an edit distance threshold of 2 was applied (Fig. 3B, left). This analysis validates that our co-evolution platform progressively enriched communities of discrete recognition clusters. When sequences from round 7 were mapped with edit distance threshold 1, the sequences formed two large, disconnected groups and several smaller clusters (Fig. 3B, right). Several notable Z-A sequences were colored in the sequence similarity network. This revealed that the nodes with the same Z-A but differing Z-B were closely connected in the same cluster, and closely related Z-A sequences which differ by one amino acid could be clustered either together (e.g., VFLV and IFLV) or separately (e.g., LVLV and LVLF). The specificity similarity network (SpSN) of Z-A sequences which connects nodes when two Z-A sequences have common Z-B partners was illustrated, and the Z-A sequences that are clustered closely in the sequence similarity network were also closely connected in the specificity similarity network (Fig. S3). For example, VFLV, LVLV, and IFLV, clustered together in a big group in the SSN, are also closely connected in the SpSN, and LVLF is clustered separately in both the SSN and the SpSN (Fig. S3). This implies that the sequence similarity network can capture the specificity of Z-A sequences from our coevolutionary sequence data. The cluster graphs, which merge each clustered community into a single node, can efficiently show such relationships between co-evolved mutants and the structure of coevolutionary networks throughout the different screening rounds (Fig. S4). Collectively this network level analysis reveals the extreme sensitivity of the specificity and cross-reactivity properties of Z-A and Z-B proteins to even single amino acid changes.

In addition to the SSN, we utilized another visualization method to depict the cross-reactivity profiles of the NGS data in our coevolutionary libraries. The Circos plot shows the pairwise relationships, highlighting the relative cross-reactivity and orthogonality of the Z-A and Z-B proteins in both LL1 and LL2 libraries (Fig. 3C and Fig. S5-S6). We sampled 100 representative pairs to present in the plot, normalizing each pair to equal area in order to visualize the approximate cross-reactivity of each sequence. A series of Circos plots spanning all screening rounds (naïve, R2, R4, R5, R6, R7, and R8) reveals the progressive shifts in cross-reactivity during the selection process. For example, we observe the emergence of poly-specificity among certain dominant Z-A sequences and increased cross-reactivity between sequences in later rounds of selection in both the LL1 and LL2 libraries (Fig. S5 and S6). This result illustrates a broad range of specificity and orthogonality within our library.

We next attempted to track the mutational pathways of specific coevolved pairs to assess how these dimer interfaces were diversified along the course of coevolution. We generated single mutational evolutionary pathways connecting the original sequence (QFLI/LVIF) with the prominent LL2 library mutants (Fig. 3D). First, we traced the Z-A pathway from the cluster graphs to identify the connected intermediates starting from the original sequence (QFLI) to the late mutants (LVFF, IVFF) (Fig. S7). The connectivity between early mutants (QFLI-VFLI), mid mutants (VFLI-VFLV-VFLF-VVLF-LVLF), and late mutants (LVLF-LVFF-IVFF) can be visualized from cluster graphs at different screening rounds (Fig. S7B). The ability to trace mutational pathways suggest this platform could be useful for simulating natural protein-protein evolution trajectories.

To investigate the structural energetic mechanism mediating the changes in specificity during coevolution, we measured thermodynamic binding signatures by performing isothermal titration calorimetry (ITC) of several Z domain-affibody pairs along an evolutionary pathway (Fig. 3E, S8A, and S9). We see clear evidence for enthalpy-entropy compensation over the course of coevolution, and a trend where early strongly favorable enthalpy and unfavorable entropy transition to produce a less favorable binding enthalpy compensated by a more neutral entropy (Figs. 3E, S8). For example, we sampled representatives from the LL2 mutational pathway from the ‘founder’ pair (QFLI/LVIF) to IVFF/FILV (Figs. 3D, 3E). Although the overall free-energy landscape of this trajectory is flat, we see changes when examining the entropic and enthalpic terms. Binding of VFLV/IVVY and LVLF/FIIV are highly enthalpically favored and entropically disfavored, but by the end of the trajectory we see a more moderate enthalpy of binding coupled with a moderately disfavored entropy in IVFF/FILV. Although we could not observe any structural features that distinguish cross-reactive versus selective complexes, the thermodynamic properties of cross-reactive mutants (A-VFLV and A-LVLF) and specific mutant (A-IVFF) differed. We also followed a single mutational three-step evolutionary trajectory from LILFK/FIVM to LIFFK/FILF which are the two high affinity orthogonal pairs from LL1 library showing similar thermodynamic trends (Fig. S8A). A dramatic thermodynamic transition occurs when Leu17^A^ was mutated to Phe, to produce a less favorable binding enthalpy compensated by a more neutral entropy in specific mutant (LIFFK/FILF). Phenylalanine is often conserved in protein-protein binding sites, and aromatic residues frequently serve as anchor residues to mediate protein-protein interactions (22). The common mutation in both mutants, Leu17^A^Phe, may act as a new anchor residue, thus leading to more entropically favored interactions between two proteins (23).

We next verified by cleavage-capture assay the relative specificities of each Z-A sequence toward Z-B sequences from this evolutionary pathway (Fig. 3F). Starting from the early mutants, the specificity matrix clearly indicates gradual and continuous compensatory changes of binding preference between variants along the mutational pathway for both LL1 and LL2 libraries (Fig. 3F and Fig. S8B). Thus, we could systematically track the diversification of specificities and cross-reactivities within our library by mapping of the coevolutionary network.

### Direct coupling analysis and structural adaptations in coevolved complexes

**Figure 4.**
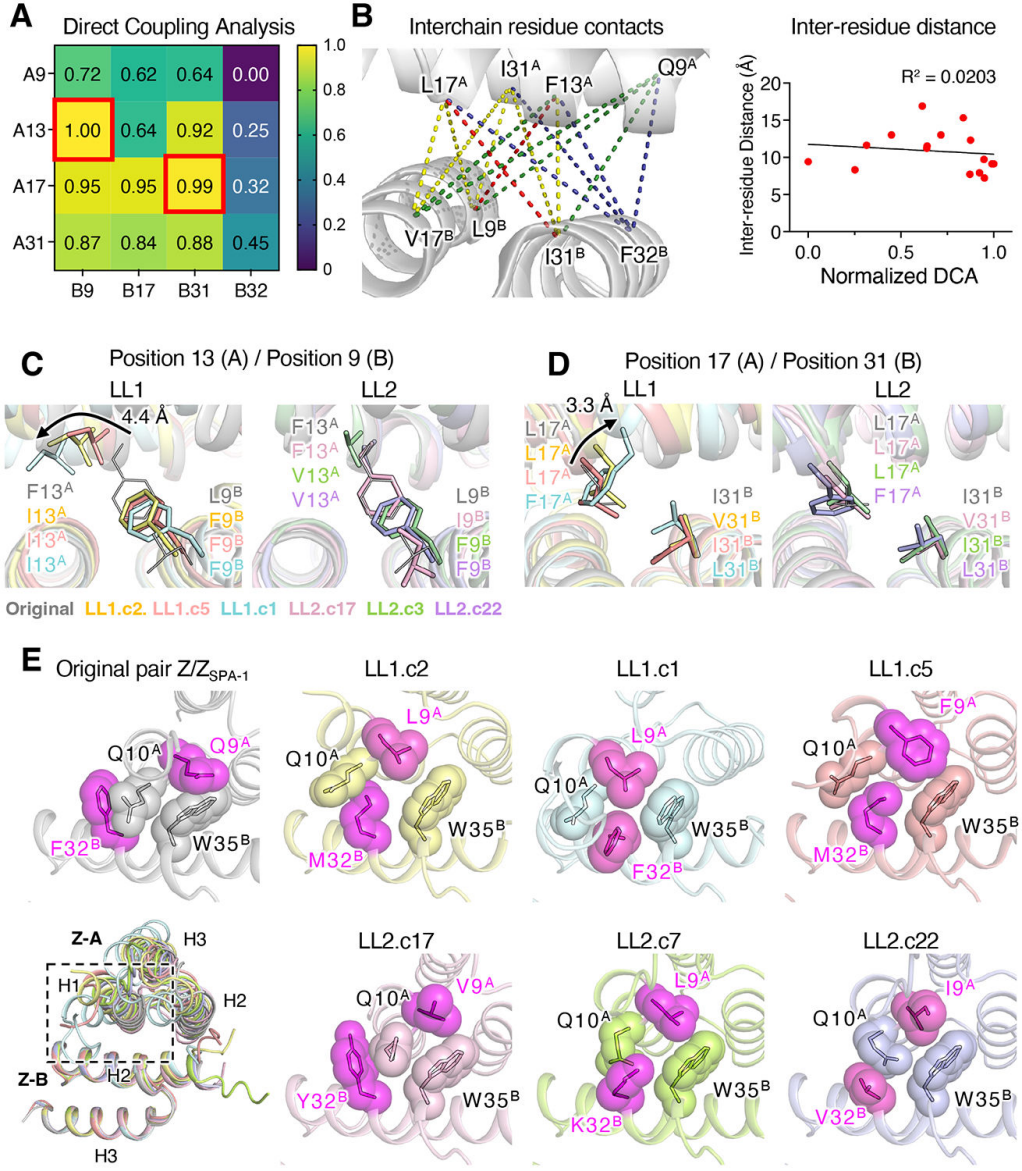
Coupling analysis and structural adaptation of coevolved variants. (A) DCA matrix to predict inter-residue covariation of LL2 library sequences (round 6 and 7). The DCA scores are normalized between 0 and 1. The pairs with the highest DCA scores, 13^A^-9^B^ and 17^A^-31^B^, are marked with red squares. The matrix rows represent residues from Z-A, columns represent residues from Z-B, and the elements represent the statistical dependencies between residues. Through the inverse covariance matrix analysis, the pairs 13^A^-9^B^ and 17^A^-31^B^ were identified as strongly interacting pairs, indicating their direct contact in the 3D structures. (B) Inter-residue contacts (left), and the relationship between DCA and inter-residue distance is measured from the original pair structure (right) (PDB: 1LP1). The dashed lines are color-coded (from purple to yellow) based on DCA matrix in panel (A). The top two highest DCA contacts (Leu 17^A^ – Ile 31^B^, Phe 13^A^ – Leu 9^B^) are colored in red. The overall relationship between inter-residue distance and DCA score was weak (R^2^ = 0.0203). (C-E) Close-up views of library positions to show local side chain rearrangements. Pairs of residues at the center of the dimer interface were mutated in a compensatory manner between 13^A^ and 9^B^ (C) and between 17^A^ and 31^B^ (D). Side chain substitutions from 4 different interacting pairs are shown as sticks (E) Library positions 9^A^ and 32^B^ are closely associated with proximal residues, Gln10^A^ and Trp35^B^, maintaining the shape complementarity between two proteins. In the bottom left, B chains of seven interacting pairs are aligned, with close up views of the boxed region shown for each pair. Coupled side chains are shown as sticks with transparent spheres to indicate packing interactions.

We sought to evaluate the accuracy of coevolutionary patterns between residues in predicting protein interaction contacts (Fig. 4). The coevolution of residues in protein sequences is affected by epistatic couplings, which may or may not match with structural contacts (24). First, we used mutual information (MI), a measure of the statistical coupling between any two positions in a protein pair, which can reflect structural interactions. To do this, we again filtered protein pairs to statistically enrich for those pairs occurring significantly more often than expected, mirroring our approach in the SSN analysis (see Methods). Using these filtered pairs, we calculated pairwise MI between all residues in the LL1 and LL2 screens (Fig. S10). MI serves as a local information theoretical metric, enabling us to determine the level of dependence between two positions. Our results showed that the top-ranked inferred coupling (17^A^-31^B^) was consistent with known contacts in the 3D structure of the original pair, indicating that structural constraints are captured in the sequence coevolution. Next, we applied a direct coupling analysis framework to the *unfiltered* LL2 sequences, which constitute a larger and more complex library (25). Our goal was to determine if direct interactions could be inferred with the increased size and complexity of the LL2 library and a global statistical method. We used the inverse covariance matrix to infer direct contacts. The columns in the matrix represent residues from one protein, rows represent residues from another protein, and elements represent the statistical dependencies between residues (Fig. 4A). By analyzing the inverse covariance matrix, we identified 13^A^-9^B^ and 17^A^-31^B^ as strongly interacting pairs, which supports their direct contact with each other in 3D structures. The top 5 highly correlated residues were close in the original structure, but the overall relationship between inter-residue distance and DCA score was weak (Fig. 4B).

To clarify these inter-residue co-variations discovered from the sequence data, we determined crystal structures of 10 coevolved pairs that spanned a range of cross-reactivities and orthogonalities (5 from LL1 and 5 from LL2). All structures were solved at high resolution (ranging from 1.00 to 1.92 Å resolution) (Fig. S11-S13 and Tables S1 and S2). From the structures, we could verify clear compensatory changes between the residues showing the most significant covariations (13^A^-9^B^ / 17^A^-31^B^) in both LL1 and LL2 library mutants. Phe13^B^ of the Z domain, which is a core residue of the central hydrophobic patch in the original dimer, was mutated to the smaller Ile or Val in both LL1 and LL2 library mutants, and this was compensated by mutation of the opposing residue Leu9^A^ to the larger Phe (Fig. 4C). We also observed another highly correlated opposing residue pair (17^A^-31^B^) mutated in a compensatory manner in both libraries. Interestingly, here Leu17^A^Phe is rotated outward, accommodating Ile31^B^Leu to fill the cavity between the two proteins (Fig. 4D).

The extent of interface structural remodeling in all complexes due to the coevolution selection pressure is made clear in Fig. 4E, where the non-mutated residues Q10^A^ and W35^B^ accommodate the library mutations at positions 9^A^ and 32^B^ by adopting completely different positions and local environments (Fig. 4E). The two library positions (9^A^-32^B^) and proximal residues, Gln10^A^ and Trp35^B^, kept close contact in all mutant structures, albeit with different interactions. The ability to rearrange at these positions allows decoupling of mutations despite close proximity (Fig. 4E). These results indicate that the protein interfaces of both specific and cross-reactive complexes were completely remodeled in different ways to improve affinities up to three logs (K_D_ of LL1.c2 = 1.86 nM, original = 2.92μM) and bias specificities (K_D_ of Z-A^LL1.c4^ (FILFK) with Z-B^LL1.c4^ (FIVM) = 2.53 nM, and with Z_SPA-1_ (LVIF) = 21.9μM).

### Cross-reactivity and orthogonality in coevolved dimer structures

**Figure 5.**
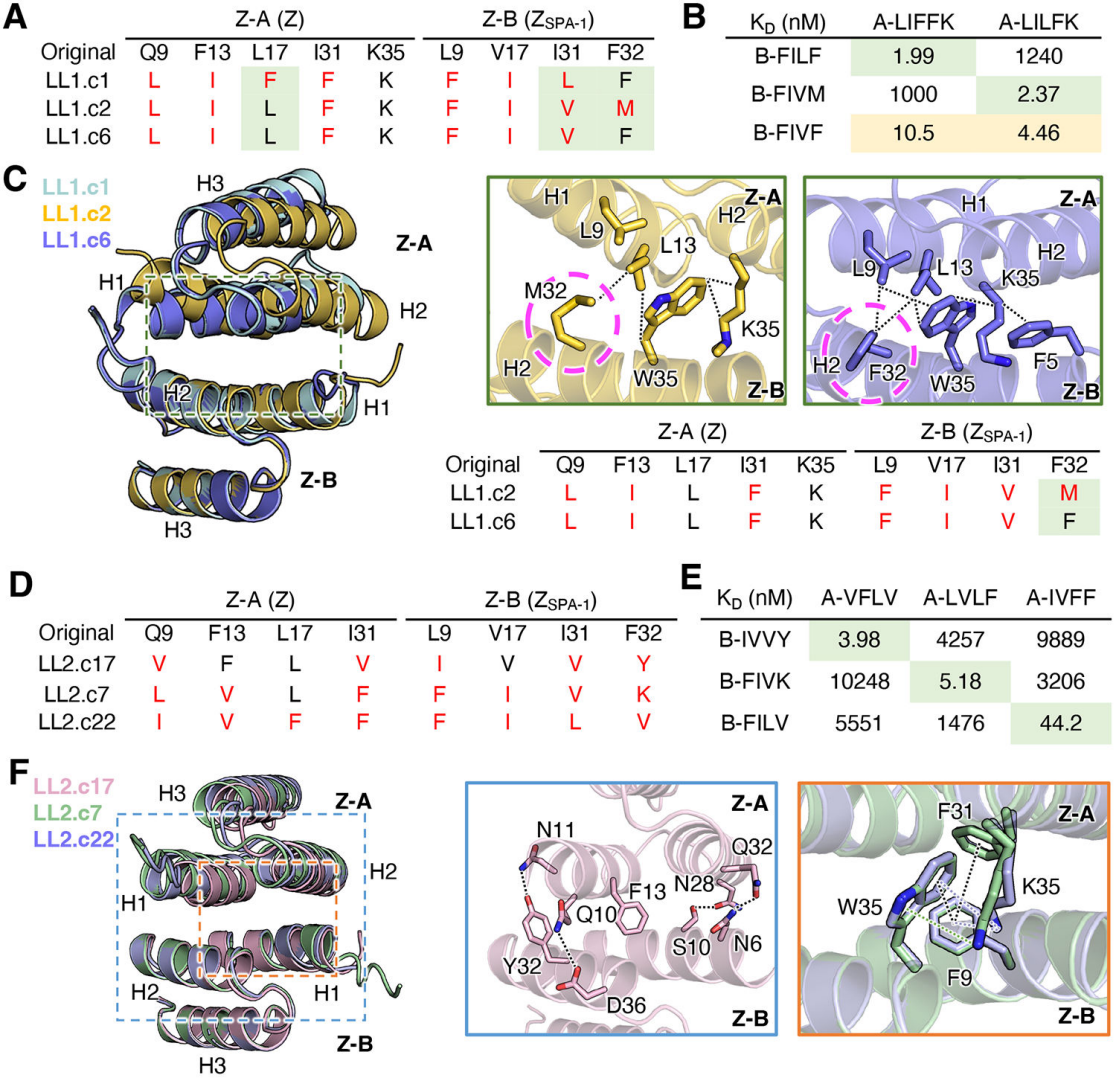
Specificity determinants of orthogonal high-affinity mutants. (A) The altered positions compared to the original amino acids are colored red, and varying positions between mutants are highlighted with green boxes. (B) A table of affinity between Z-A and Z-B monomers measured by SPR. LL1.c1 and c2 are orthogonal to each other and B-FIVF of LL1.c6 are cross-reactive to both Z-A mutants. (C) Comparison of LL1.c2 and LL1.c6 structures near position 32^B^ shows how the single mutation M32^B^F induces large conformational changes by side chain rotation of Trp35^B^ and increased hydrophobic interactions around it. Superposition of overall structures of LL1.c1, LL1.c2 and LL1.c6 (left). Close-up views of each mutant show Trp35-centered hydrophobic interactions with surrounding residues (right). Position 32 is highlighted with dashed circles. (D) A table showing amino acids in library positions of the three orthogonal LL2 mutants, LL2.c17 (VFLV/IVVY), LL2.c7 (LVLF/FIVK) and LL2.c22 (IVFF/FILV), that were selected to compare differences in their affinity and structures. (E) Binding affinities of each combination of Z-A and Z-B mutants of the three mutants. (F) Significant structural difference at the interface of LL2.c17 and other two mutants. Superposition of overall structures (left). Close-up views of interface (right). LL2.c17 has Phe13^A^ as the core of a central hydrophobic patch surrounded by multiple hydrogen bonds. LL2.c7 and c22 have a Phe9^B^-centered hydrophobic patch composed of clustered pi-pi interactions and cation-pi interactions (F31^A^, K35^A^, F9^B^, and W35^B^).

The availability of a large panel of coevolved mutants allows us to ask questions about their relative cross-reactivity versus specificity. For example, A-LILFK has more Z-B binding partners (B=77) than A-LIFFK (B=15) from the LL1 library, and A-LVLF (B=53) and A-VFLV (B=42) are also more cross-reactive than A-IVFF (B=3) from LL2 library sequence data. To answer the question of what causes differences in the cross-reactivity of certain Z-A sequences and to clarify specificity-determining residues, we compared high-affinity mutant structures of each Z-A sequence (Fig. 5). First, the binding preferences of the two highest affinity pairs from the LL1 library, LL1.c1 (LIFFK/FILF) and LL1.c2 (LILFK/FIVM), are nearly completely orthogonal, so we focused on investigating specificity-determining positions of the two variants (Fig. 5A and 5B). The two mutants differ by only three amino acid positions (positions 17^A^, 31^B^, and 32^B^) but have virtually no cross-reactivity with each other. Z-A sequences of LL1.c1 and LL1.c2 bind to their own Z-B sequence 500-fold stronger than when mixed with the other’s Z-B sequence (Fig. 5B). On the other hand, B-FIVF, which has only a single amino acid change from B-FILF or B-FIVM, has poly-specificity and binds to both Z-A sequences (A-LIFFK and A-LILFK) with moderate affinity (Fig. 5B). Thus, the mutant LL1.c6 (LILFK/FIVF) can be a “bridging” intermediate to help explain the structural evolution of orthogonality through cross-reactivity. Comparing structures of LL1.c2 and LL1.c6 revealed that the single mutation Met32^B^Phe in LL1.c6 induced a noticeable geometric change by forming an enhanced hydrophobic cluster within the binding interface (Leu9^A^, Leu13^A^, Lys35^A^, Phe5^B^, Phe32^B^, and Trp35^B^) (Fig. 5C). Due to the rotation of Trp35^B^ and Trp35^B^-centered hydrophobic packing induced by Phe32^B^, the N-terminal end of helix 1 and C-terminal end of helix 2 of Z-A tilted 17° closer to Z-B. Furthermore, the compensatory relationship between position 17^A^ and position 31^B^ is clearly revealed from the structures of LL1.c1 and LL1.c2 (Fig. 4D). Taken together, the synergistic effects of the geometric change and compensatory mutations found from these three specificity-determining positions resulted in biased specificities of the two high-affinity variants evolved from the same library.

The monomers from the high affinity mutants from the LL2 library (three Z-A mutants and five Z-B mutants) are even more orthogonal to one another (Fig. S2F and S2G). The three orthogonal mutants, LL2.c17 (VFLV/IVVY), LL2.c7 (LVLF/FIVK) and LL2.c22 (IVFF/FILV), were selected to compare differences in their affinity and structures (Fig. 5D). Each Z-A mutant binds to its binding partner with nanomolar affinity (3.98 to 44.2 nM) but has minimal cross-reactivity with other monomers (Fig. 5E). The backbone structures of the three mutants are relatively similar (Cα r.m.s.d. of Z-A after aligning Z-B ranges from 0.447 Å to 0.641 Å). The dimer interactions of LL2.c17 have sharply diverged from the other two LL2 mutants, with the Phe13^A^-centered hydrophobic patch surrounded by multiple rewired hydrogen bonds, an additional hydrogen bond between Asn11^A^ and Phe32^B^Tyr, and α-helix 2 of Z-A was slightly shifted to generate new interactions with helix 1 of Z-B, which explains the significantly improved affinity between these two proteins compared to the original pair (Fig. 5F and S14). The other two mutants, LL2.c7 and c22, have clustered pi-pi and pi-cation interactions at the interface (Phe31^A^, Lys35^A^, Phe9^B^, and Trp35^B^) (Fig. 5F). Additionally, the same compensatory mutations (positions 17^A^ and 31^B^) as LL1 mutants are also seen from LL2.c7 and c22 mutants (Fig. 4D). Contrary to the LL1 library, LL2 library mutants have less dramatic change in backbone orientation, but interfaces are more diverse due to the broader amino acids available to be mutated in library positions.

We do not observe systematic differences in the structural parameters of the interfaces mediating specific (A-LIFFK in LL1 and A-IVFF in LL2) versus cross-reactive (A-LILFK in LL1 and A-VFLV, A-LVLF in LL2) complexes. All mutants except for one (LL2.c1) had a higher fraction of nonpolar BSA than the original dimer (58%), and all mutants except for one (LL2.c7) had a higher packing score (PackStat) than the original dimer (PackStat of Z/Z_SPA-1_ = 0.640) (Table S3). The cross-reactive complexes did not show evidence of poorly packed interfaces, non-ideal bonding that might predispose them to promiscuity; in this sense they are indistinguishable from protein interfaces of the specific complexes.

### Using protein language models to predict novel dimer interactions from co-evolved protein sequences

**Figure 6.**
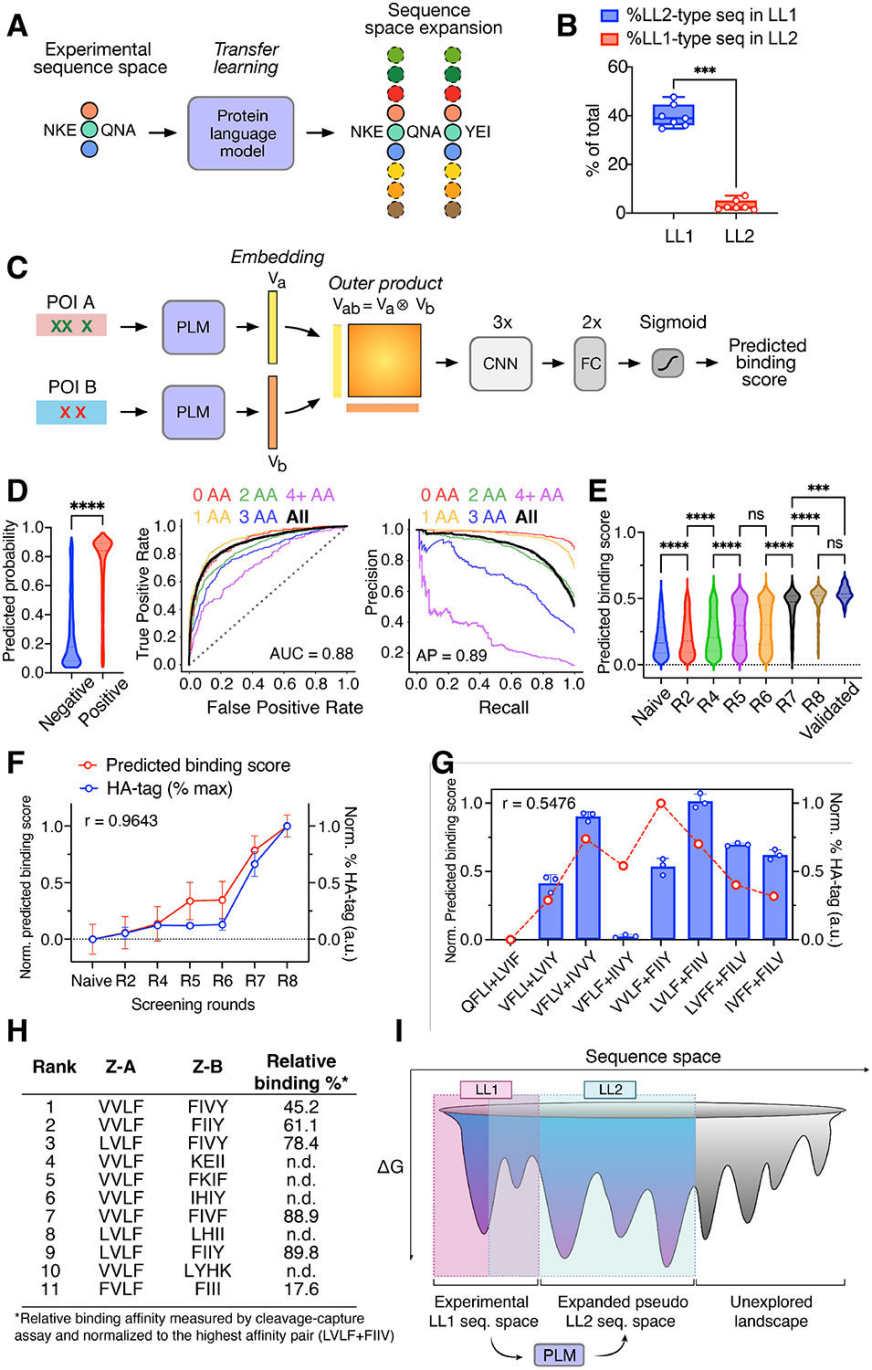
Sequence space expansion using protein language model (A) A schematic representation of sequence space expansion through protein language model. (B) The fraction of LL1-type sequences (the Z-A and Z-B sequences can be encoded with LL1 degenerate codon sets) in LL2 sequencing data and vice versa. Fractions of each screening round (from naïve to R8) were represented in a Box plot with individual data points. A two-tailed Mann–Whitney test was used to analyze results. *** *P* < 0.001. (C) A schematic representation of our approach to predict dimer interactions with expanded set of amino acids using outer product-based convolutional neural network. (D) The classification efficiency of LL1-trained model on LL2 test set. (left) A violin plot representing predicted binding score of negative (*n* = 2,771) and positive (*n* = 2,794) data. Two-tailed Mann–Whitney test. **** *P* < 0.0001. (middle) A ROC plot and (right) a PR plot. Note that the sequences in test set were categorized into five groups based on the number of new amino acids compared to the LL1 sequence data, allowing an assessment of the impact of dissimilarity between the two libraries on predictions. The AUC (Area Under the ROC curve) and AP (Average Precision) values of total sequences and each subgroup are: all sequences (*n* = 5,565, AUC = 0.88, AP = 0.89), 0 AA (*n* = 508, AUC = 0.88, AP = 0.98), 1 AA (*n* = 1,332, AUC = 0.91, AP = 0.97), 2 AA (*n* = 1,509, AUC = 0.84, AP = 0.87), 3 AA (*n* = 1,091, AUC = 0.80, AP = 0.70), 4 and more AA (*n* = 1,125, AUC = 0.73, AP = 0.32). The diagonal dotted line in ROC plot represents AUC = 0.5. (E) The predicted binding scores of LL2 sequencing data of each screening round were represented in a violin plot. One-way ANOVA. ***P < 0.001, ****P < 0.0001. ns, not significant. (*n* = 28–10,000) (F) The correlation between predicted binding score of LL2 sequencing data and actual %HA-tag MFI after protease cleavage. Normalized %HA-tag MFI and predicted binding score of each round was compared by Spearman’s correlation test (*r* = 0.9643, *P* = 0.0028). Data are mean ± SD; n = 3 independent replicates for HA-tag MFI measurements. (G) The correlation between predicted binding score and relative affinity of the pairs from the mutational pathway in Fig. 6A. Normalized % of max HA-tag MFI from cleavage-capture assay and predicted binding score of each round was compared by Spearman’s correlation test (*r* = 0.5476, *P* = 0.0855). (H) Top 11 sequences by predicted binding score from LL2 NGS data. The binding of 6 out of the 11 sequences were verified by on-yeast cleavage-capture assay and their relative binding affinities were normalized to the high affinity LL2 pair, LL2.c3 (LVLF+FIIV). n.d. = not detectable affinity by the assay. (I) A cartoon representation depicting the expansion of sequence space from experimental LL1 data to the predicted LL2 sequence space using a protein language model and transfer learning.

The large database of coevolved complexes led us to ask if this information could be used to inform predictions through machine learning. One limitation of our experimental screen was that we used a limited set of amino acid codons in our experimental screen in order to fully sample the diversity of the yeast display libraries. But this raised the question of how to predict the binding affinity of larger diversity libraries containing more diverse amino acids without exceeding the practical diversity limits of the screening platform. One solution is the use of protein language models, which are self-supervised machine learning models pre-trained on large protein sequence databases (15, 26-28). We used protein language models to expand the set of amino acids in our screen through the process of transfer learning (Fig. 6A). Transfer learning involves applying knowledge gained from one problem to solve a related problem. By using a common protein language model to embed pairs of protein sequences, we can learn complex patterns that predict protein-protein interactions using a limited set of amino acids and then apply this knowledge to predict binding affinity for novel pairs using a broader set of amino acids. Our two coevolution libraries, LL1 and LL2, used different subsets of amino acids to mutate library positions and yielded differently enriched sequences after screening (Fig. 2). The LL2 library (11 AA) has an expanded amino acid diversity compared to the LL1 library (8AA for Z-A and 5AA for Z-B), and only 3.2% of LL2 library sequencing data is LL1-type sequences (compatible with LL1 degenerate codon sets) while LL1 data has 40% of LL2-type sequences on average (Fig. 6B). Therefore, these two libraries are appropriate model systems to test how LL1 sequence data trained model can expand sequence space and predict new interactions only possible from LL2 sequence data.

To determine if protein language models could be used to model Z-affibody pairs in our screens, we used the pre-trained ESM protein language model (16), which incorporates knowledge of all amino acids from large evolutionary sequence datasets, to generate embeddings of the sequences observed in LL1 (Fig. 6C). We predict dimer interactions using the outer product of individual protein sequence embeddings, and the resulting outer product matrix is then used as input into a convolutional neural network for further processing. This approach allows us to model complex interactions of protein-protein interface sequences, including those featuring an expanded set of amino acids than those used in our experimental screen.

We first encoded the individual protein sequences from each protein pair in the LL1 screen and trained a deep neural network to identify positive interacting pairs. Positive interactions were defined as enriched or filtered protein pairs occurring significantly more often than expected in the enriched library (rounds 6 and 7), reflecting our methodology in the SSN and DCA analyses (see Methods). Negative interactions were defined as protein pairs that were present in the naïve library NGS data but absent in rounds 6 and 7. Given that each round captured 7-10 times more cells than the observed diversity of sequences in the naïve library, we reason the absence of these interactions is most likely due to being outcompeted during co-evolution. Next, we sought to evaluate the performance of the LL1-trained model in classifying held-out positive and negative LL2 interactions, which contain an extended amino acid library (Fig. 6D). The LL1-trained model could classify 5,565 LL2 sequences in our held-out test set (2,794 positive and 2,771 negative) with AUC of .88 (Fig. 6D). We then specifically examined the LL1-trained model’s ability to generalize by assessing its capacity to handle a progressively expanding amino acid library. We binned the LL2 test data based on the number of previously unused amino acids incorporated in the held-out test sequences (0, 1, 2, 3, 4 or more) relative to the LL1 sequence training data. These amino acids, which were not included in the LL1 training library, serve as indicators of the difference between the test and training sequences. Despite a decrease in performance trend as more amino acids are introduced, the LL1-trained model still achieves an AUC of 0.8 and an AP of 0.7, even when up to 3 out of 8 amino acids are not part of the LL1 training library. These tests demonstrate the model’s robustness in handling sequence variations and making reliable predictions.

Finally, we applied our LL1-trained model to all LL2 screening rounds from naïve to final round 8 to assess the ability of the model to predict interactions of pairs in different selection stages (Fig. 6E). The predicted binding scores of each round increases as screening proceeds, and the mean of the predicted scores at each round is highly correlated with actual %HA-tag fluorescence level after protease cleavage (Fig. 6E and 6F). We also compared the predicted scores with experimentally validated pairs in Fig. 3. The 28 validated interacting pairs (% of max HA-tag > 1) in Fig. 3G showed elevated predicted binding scores (Fig. 6E). The model could even moderately predict affinity changes between pairs along the mutational pathway in Fig. 3E (Fig. 6G). Even though two intermediates (VFLF+IIVY and VVLF+FIIY) were predicted to have higher affinities than their actual affinities, overall trends are similar between prediction and affinity throughout the pathway. We also evaluated the accuracy of our model in identifying hits among the top-ranked sequences. We conducted experimental validation on the binding of the 11 highest-ranked sequences and found that 6 out of the 11 sequences (hit rate = 54.5%) demonstrated affinities within the detectable range (submicromolar) as confirmed by the on-yeast cleavage-capture assay (Fig. 6H). These data demonstrate that we can use a protein language model to expand sequence space from the experimental sequence data of LL1 and predict the new interactions that we observed from LL2 screening data (Fig. 6I).

## Discussion

We have developed a facile method for protein-protein coevolution as a solution to the problem of linking phenotype to genotype in large-scale library-on-library selections (29-31). The large collection of interacting Z-domain/affibody pairs we generated enabled a systems-level structure-function analysis of molecular recognition within this model system. We observed important characteristics of natural protein-level coevolution, including compensatory mutations between residues and hydrophobic core repacking. Acquiring compensatory mutations between directly interacting proteins is the simplest molecular mechanism that can cause epistasis between two genes (32, 33). Based on direct coupling analysis and high-resolution crystal structures, we could successfully infer epistatic interactions between Z domain-affibody dimer interfaces. The crystal structures of coevolved mutants revealed that when a key residue of the original central hydrophobic patch, Phe13^A^, was mutated to smaller amino acids like leucine or valine, Phe9^B^ or Trp35^B^ newly form the core of the central hydrophobic patch, presumably rearranging an existing hot spot or creating new ones. We infer that coevolving contact residues can fundamentally change binding interfaces to have different specificities and affinities by reinforcing or rearranging hot spots. The remodeling of the dimer interface of the Z domain and affibody was similar to the repacking of the hydrophobic core of widely-studied proteins such as Rop, T4 lysozyme, and λ Repressor-GCN4 Leucine Zipper Fusions (34-38). Thus, interface coevolution appears to follow principles of protein core repacking (39-42).

In the course of our experimental studies of coevolution we identified a challenge in the form of a curse of dimensionality, where an exponential increase in experimental data is needed to test protein interactions as the number of mutating positions and amino acid alphabet increases. This issue is a major practical limitation to protein engineering using combinatorial libraries because full diversity libraries exceed the experimental diversity possible in yeast, phage, or ribosome display. To address this challenge, we used protein language models. Previously, protein language models have been limited to predicting monomeric properties, and fine-grained variant effect analysis of protein-protein interactions has been difficult to evaluate due to a lack of data. Here, we demonstrate that by leveraging a shared sequence space learned from large-scale protein sequence databases, we can both extract informative representations of protein sequences and model their binding interactions. The amino acid composition of a protein encodes the information required to determine not just its structure but also its ability to negotiate interactions with other functional partners. Therefore, by using the information encoded in the latent protein embedding space, we can explore a larger space of protein-protein interactions than what is experimentally available. This approach combined with transfer learning can reduce data requirements and provide reliable predictions of binding interactions.

This synthetic coevolution strategy can potentially be used in biotechnology applications. Although AlphaFold and RoseTTAFold are useful for predicting 3D protein structures from the amino acid sequence, predicting de *novo* protein-protein interactions remains a challenge (43). The experimental data generated from our coevolution strategy can be used as training data for machine learning algorithms to expand sequence space much wider than what can be obtained experimentally and to predict protein-protein interactions. The one-pot production of a large set of protein pairs with different specificity and cross-reactivity is also useful for synthetic biology. Orthogonal interfaces are essential components to build reliable and predictable orthogonal gene circuits to avoid undesirable crosstalk with the host or other machinery (44, 45). Our synthetic coevolution strategy can generate user-designed orthogonal protein complexes for such applications.

## Materials and Methods

### Protein expression and purification

The DNA plasmids encoding for each affibody were cloned into pET28, a bacterial expression vector. The vector includes the affibody gene with either only C-terminal His6-tag or biotin-acceptor peptide tag (BAP tag, GLNDIFEAQKIEW) followed by His_6_-tag between the *NcoI* and *XhoI* sites of pET28b (Novagen). To express affibody monomers, the vector was transformed into *E. coli* BL21 (DE3), and the cells were grown at 37°C in TB medium supplemented with 50mg/l kanamycin. At 0.6 OD_600_, 0.5mM isopropyl-β-D-thiogalactoside (IPTG) was added to induce protein expression and the cell culture was incubated for overnight at 30°C before harvest. The proteins were purified by Ni^2+^-NTA agarose column chromatography (Ni-NTA, Qiagen) followed by size-exclusion chromatography with a Superdex S75 10/300GL Increase column (GE Healthcare). The proteins were stored in HEPES buffered saline (HBS, 20mM HEPES pH 7.5, 150mM sodium chloride). Affibody proteins used for surface plasmon resonance experiments were site-specifically biotinylated at the C-terminal BAP tag using *BirA* ligase and re-purified by size-exclusion chromatography.

### Yeast display of single-chain Z domain-affibody dimers

Single chain affibody dimers were displayed on the surface of yeast *S. cerevisiae* strain EBY100 (Invitrogen, cat. no. C839-00) by fusion to the C-terminus of the Aga2 protein. Affibody dimers connected with a GS-linker and 3C protease cleavage site in the middle were inserted between an N-terminal cMyc epitope and a C-terminal HA tag. N-cMyc-ZA-linker-ZB-HA-C insert was cloned into the pCT302 vector (Addgene #41845). Competent yeast cells were electroporated with affibody plasmids and recovered in YPD (Sigma, cat. no. Y1375) at 30°C for an hour. Next, recovered cells were grown in SDCAA media (pH 4.5, 20 g dextrose, 6.7 g yeast nitrogen base, 5 g bactocasamino acids, 10.4 g sodium citrate and 6.4 g citric acid monohydrate dissolved in 1 liter of deionized H_2_O, supplemented with 10 ml of Gibco^™^ Penicinillin-Stereptomycin, 10,000 U/ml) to OD_600_ 10, and the cultures were induced at 20°C for 24 hours by diluting to OD_600_ 1.0 in SGCAA (prepared as SDCAA, but use 20g galactose instead of dextrose) (7). The display level of proteins was confirmed by staining the cells with an Alexa Fluor 488-labeled anti-cMyc antibody (Cell Signaling Technology, cat. no. 2279S) and Alexa Fluor 647-labeled anti-HA antibody (1:50 dilution; Cell Signaling Technology, cat. no. 3444S), and fluorescence was monitored by flow cytometry (Beckman Coulter, CytoFLEX).

### Yeast displayed libraries

Details of library assembly, sequences, and selection protocols are provided in Supplementary Methods.

### On-yeast cleavage-capture assay

For single clone cleavage-capture assay, colonies were picked from transformed EBY100 cells plated on SDCAA plate. 5 × 10^5^ induced yeast cells were stained with an Alexa Fluor 488-labeled anti-cMyc antibody and Alexa Fluor 647-labeled anti-HA antibody (1:50 dilution). Antibody-stained cells were washed with MACS buffer (autoMACS^®^ Running Buffer, Miltenyi, cat. no. 130-091-221), then incubated in 20 μL 3C protease cleavage solution (lab-made 3C protease was diluted to 0.4 mg/mL in MACS buffer) at 4°C. At each time point, 2 μL was sampled and diluted in ice-cold 100μL MACS buffer, and fluorescence was measured by flow cytometry. The measured mean fluorescence intensity (MFI) was divided by MFI before cleavage to gain % of max MFI to represent an affinity between two interacting proteins.

### Cross-reactivity Circos Plots

Circos plots were created via the circlize software package (48). In short, sequences with p-value < 0.05 were combined into separate data sets for LL1 and LL2 and further separated by screening round. A cross-reactivity score was calculated for each unique Z-A sequence by determining the number of its unique Z-B pairs per data set. Cross-reacitvity scores were then normalized to sum to 1. Finally, to facilitate visualization via circos plots, the data set was subsetted using the ‘train_test_split’ function of the python scikit-learn (version 1.2.2) package. To maintain the proportion of Z-A cross reactivity, the ‘stratify’ option was applied to the cross-reactivity score.

### Sequence Similarity Network, cluster graph and Specificity Similarity Network

Sequence similarity networks and cluster graphs were created via the igraph software package (49). Nodes of the edit distance-based networks correspond to unique Z-A/Z-B pairs. Connections are present between nodes for instances in which the edit distance of two Z-A/Z-B pairs is below a given threshold. Nodes of the Specificity Similarity Network correspond to unique Z-A sequences and connections are drawn between Z-A sequences when Z-A sequences share common Z-B sequences numbering above a certain threshold.

### Mutual Information

To measure the coevolution relationship among interface residues, we computed the mutual information (MI) between two positions i, j as $MI_{ij}=\sum_{AB}f(A_{i},B_{j})log(\frac{f(A_{i},B_{j})}{f(A_{i})f(B_{j})})$ following (Dunn et al., 2005) (48), where $f(A_{i},B_{j})$ is the observed frequency of the amino acid pair (A,B) at position i, j, $f(A_{i})$ is the observed frequency of amino acid A at position i, and $f(B_{j})$ is the observed frequency of amino acid B at position j.

### Inverse covariance matrix

To uncover direct coupling signals from the MSAs, we used a method based on the estimation of the inverse covariance matrix following (Jones et al., 2012) (25). For position i, j and amino acid pair A, B we compute the empirical covariance matrix as $S_{ij}^{AB}\;=\;f(A_{i}\;,B_{j})-f(A_{i})f(B_{j})$ where $f(A_{i}\;,B_{j})$ is the observed frequency of amino acid pair A, B at position i, j. $f(A_{i})$, $f(B_{j})$ are the observed frequency of amino acid A at position i and the observed frequency of amino acid B at position j respectively. Then we use the Graphical Lasso to estimate the inverse covariance matrix θ by maximizing the objective function $log(det(\theta ))\;-\;\sum_{ij=1}^{d}S_{ij}\theta_{ij}$ subject to the constraints $\sum_{ij=1}^{d}|\theta_{ij}|\leq \alpha$ and $\theta \;\underline{≻}\;0$ where S is the empirical covariance matrix, θ is the inverse covariance matrix and α is the sparsity constraint parameter. We set α=1 in all of our analysis. The optimization is performed with CVXPY v1.2 package in python.

### Data

To train our deep learning model, we assembled positive and negative protein-protein pair examples from the oligopeptide pair dataset from the LL1 library. For enriched samples, we filtered the intermediate enriched library and applied the hypergeometric test described in Sequence library filter with a 0.05 p-value threshold, resulting in 14,491 pairs. For naive samples, we randomly sampled 14,471 pairs from the naive library that were not present in the intermediate enriched library. We then randomly split the data into training and validation sets with 80% and 20%, respectively. For the LL2 library, we applied the same method, resulting in 2,794 enriched and 2,763 naive samples as our test set. We also normalized the sequencing counts for our training label such that all naive samples scored 0 and all positive pairs are scored according to their observed sequencing counts then Min-Max normalized as

$$X_{s}=\frac{log(count(X)+100)-log(2)}{log(maxcount)-log(2)}.$$

Note that we added 100 base counts to all positive pairs to distinguish them from the naive pairs after normalization.

### Protein Language Model embeddings

For each oligopeptide pair, we used the full chain sequence with the corresponding amino acid in the mutant position as sequence input to the protein language model for the latent vector representation generation. The vector representation is taken as the average position-wise embedding from the last layer of the protein language model with 1,280 dimensions. For each pair, we generate the sequence embeddings for each chain separately as $V_{a}$, $V_{b}$, and the outer product is computed across the vector representation of the two respective chains as a two-dimensional matrix representation for each oligopeptide pair as $V_{ab}=V_{a}⊗V_{b}$.

### Model Architecture

We designed and implemented a 3-layer 2D CNN model with kernel size (5,5) and channel size [64,128,256] followed by a two-layer fully connected network to predict the binding score of the input oligopeptide pairs. The model takes the two-dimensional oligopeptide pair representation $V_{ab}$ as the input and outputs a scalar $P_{ab}$ as the binding score. We also apply a max pooling layer and instance norm in-between each CNN layer.

$$W^{l}=InstanceNorm(ReLU(Maxpool(2Dconv(W^{l}-1))))\;\;\text{where}\;W^{0}=V_{ab}\;\;P_{ab}=FC(flatten(W^{f}))$$

where $W^{f}$ is the output of the last CNN layer.

We also applied sigmoid transformation to the fully connected network output for scaling.


$$Sigmoid(X)=\frac{1}{1-e^{-x}}$$

### Model training and testing

All models are trained with squared L2 norm loss and the Adam optimizer with learning rate of $1e^{-4}$ on a NVIDIA 2080Ti machine for 100 epochs with the best saved checkpoint. Our implementation uses the PyTorch V1.11 compiled with CUDA 10.2.

### X-ray crystallography

Details of crystallization and structure determination are provided in Supplementary Methods along with structure statistics Tables S1-S2.

### Surface plasmon resonance

Dissociation constants (K_D_) of affibody dimers were acquired by surface plasmon resonance (SPR) using the BIAcore T100 instrument (GE Healthcare). Approximately 100 resonance units (RU) of biotinylated affibody were captured on a streptavidin-coated (SA) sensor chip (Cytiva), including a reference channel with an unrelated protein. HBS-P+ (Cytiva) was used for all SPR runs. All measurements were made with two-fold serial dilutions using 60-120 s association and 300-500 s dissociation at a flow rate of 30-50 μl/min. Regeneration was performed using 0.02% SDS or 0.1M glycine, pH 2.5 after each analyte injection. The sensorgrams obtained were either fit to the 1:1 binding model or the steady-state affinity model using the BIAcore T100 evaluation software.

### Isothermal titration calorimetry

For isothermal titration calorimetry experiments, proteins were dialyzed overnight against HBS buffer. After dialysis, concentrations were measured using the BCA assay kit (Thermo Fisher). Titrations of all mutants were performed in a Microcal VP-ITC instrument at 298 K with Z_SPA-1_ variants in the cell at 5 μM and Z variants in the syringe at 7-10× the cell concentration. The parent Z_SPA-1_ protein was used in the cell at 50 μM, with the parent Z protein in the syringe at 350 μM. Baseline subtraction was performed by titrating Z variants or Z parent into the dialysis buffer. All data were analyzed in Origin 7.0, fit to a 1-site model by fitting ΔH, K_a_, and the number of binding sites (n).

## Supplementary Material

Yang et al-Supplement
