# Supplementary material for: Deploying synthetic coevolution and machine learning to engineer protein-protein interactions
Source: Science. Author manuscript; Available in PMC 2023 Aug 4. (PMC10403280; doi:10.1126/science.adh1720)
Supplement: Yang et al-Supplement [file NIHMS1914187-supplement-Yang_et_al-Supplement.docx]

Supplementary Materials for

**Deploying synthetic coevolution and machine learning**

**to engineer protein-protein interactions**

Aerin Yang, Kevin M. Jude, Ben Lai, Mason Minot, Anna M. Kocyla, Caleb R. Glassman, Daisuke Nishimiya, Yoon Seok Kim, Sai T. Reddy, Aly A. Khan, K. Christopher Garcia

Correspondence to: kcgarcia@stanford.edu

**This PDF file includes:**

Materials and Methods

Figs. S1 to S14

Tables S1 to S3

References (50 – 68)

**Supplementary Methods**

Yeast displayed libraries

Four site-directed affibody dimer libraries (HL1, HL2, LL1, LL2) were created by assembly PCR using the DNA sequences and degenerate codons. The gel-purified assembled product was combined with linearized pCT302 vector transformed into EBY100 by electroporation (*7*). The electroporated cells were recovered in YPD at 30°C for an hour before passaging in SDCAA media. Serial dilutions of the recovered cells were plated onto SDCAA agar plates (Teknova) to titer the transformation efficiency. After 2 days, protein expression was induced with SGCAA media as described above.

Affibody dimers with linker DNA sequences are listed below.

HL1 Library:

GTTGATAATAAATTTAATAAAGAGCAACAAAACGCA**NTN**TATGAAATT**NTN**CATCTGCCCAATTTGAACGAGGAACAGAGAAACGCTTTC**NTN**CAGTCTCTAAAAGATGATCCAAGTCAATCAGCAAATTTACTTGCCGAAGCTAAGAAGTTAAATGACGCTCAAGCGCCTAAGGAATTCGGCGGAGGTGGGAGCCTGGAAGTTCTGTTCCAGGGTCCGGGAGGCGGCGGGAGCGGATCCGTTGATAACAAATTCAATAAGGAAACTCAGGAAGCCTCCTGGGAAATA**NTN**ACACTGCCTAATTTAAATGGTAGACAAGTCGCAGCGTTT**NTN**TCATCTCTT**NTN**GATGACCCAAGCCAAAGTGCCAACCTATTGGCTGAGGCAAAGAAATTAAACGATGCTCAAGCTCCCAAA

Theoretical nucleotide diversity: 1.68 $\times$ 10^7^

Functional library size: 2.63 $\times$ 10^9^

HL2 Library:

GTTGATAATAAATTTAATAAAGAGCAACAAAACGCA**NWW**TATGAAATT**NWW**CATCTGCCCAATTTGAACGAGGAACAGAGAAACGCTTTC**NWW**CAGTCTCTAAAAGATGATCCAAGTCAATCAGCAAATTTACTTGCCGAAGCTAAGAAGTTAAATGACGCTCAAGCGCCTAAGGAATTCGGCGGAGGTGGGAGCCTGGAAGTTCTGTTCCAGGGTCCGGGAGGCGGCGGGAGCGGATCCGTTGATAACAAATTCAATAAGGAAACTCAGGAAGCCTCCTGGGAAATA**NWW**ACACTGCCTAATTTAAATGGTAGACAAGTCGCAGCGTTT**NWW**TCATCTCTT**NWW**GATGACCCAAGCCAAAGTGCCAACCTATTGGCTGAGGCAAAGAAATTAAACGATGCTCAAGCTCCCAAA

Theoretical nucleotide diversity: 1.68 $\times$10^7^

Functional library size: 2.38 $\times$10^9^

LL1 Library:

GTTGATAATAAATTTAATAAAGAG**HWM**CAAAACGCA**HWM**TATGAAATT**HWM**CATCTGCCCAATTTGAACGAGGAACAGAGAAACGCTTTC**HWM**CAGTCTCTA**HWM**GATGATCCAAGTCAATCAGCAAATTTACTTGCCGAAGCTAAGAAGTTAAATGACGCTCAAGCGCCTAAGGAATTCGGCGGAGGTGGGAGCCTGGAAGTTCTGTTCCAGGGTCCGGGAGGCGGCGGGAGCGGATCCGTTGACAACAAGTTTAACAAAGAG**DTS**AGCGTTGCGGGTAGGGAGATA**DTS**ACGCTTCCGAATCTTAATGACCCACAGAAGAAAGCTTTC**DTSDTS**TCACTGTGGGATGACCCTAGCCAGAGCGCCAACCTGCTGGCTGAGGCGAAGAAGTTAAACGATGCACAAGCACCTAAA

Theoretical nucleotide diversity: 3.22 $\times$10^8^

Functional library size: 2.34 $\times$10^9^

LL2 Library:

GTTGATAATAAATTTAATAAAGAG**NWW**CAAAACGCA**NWW**TATGAAATT**NWW**CATCTGCCCAATTTGAACGAGGAACAGAGAAACGCTTTC**NWW**CAGTCTCTAAAAGATGATCCAAGTCAATCAGCAAATTTACTTGCCGAAGCTAAGAAGTTAAATGACGCTCAAGCGCCTAAGGAATTCGGCGGAGGTGGGAGCCTGGAAGTTCTGTTCCAGGGTCCGGGAGGCGGCGGGAGCGGATCCGTTGACAACAAGTTTAACAAAGAG**NWW**AGCGTTGCGGGTAGGGAGATA**NWW**ACGCTTCCGAATCTTAATGACCCACAGAAGAAAGCTTTC**NWWNWW**TCACTGTGGGATGACCCTAGCCAGAGCGCCAACCTGCTGGCTGAGGCGAAGAAGTTAAACGATGCACAAGCACCTAAA

Theoretical nucleotide diversity: 4.29 $\times$ 10^9^

Functional library size: 1.91 $\times$ 10^9^

Selection of yeast-display libraries

At first, induced naïve library was cleaned up by negative selection with 3-10 times of theoretical diversity of yeast cells in each library (6.5 $\times$ 10^9^ to 1.32 $\times$ 10^10^ cells). Then library selection was performed by alternating positive and negative selections with magnetic-activated cell sorting (MACS) to enrich interacting pairs but prevent accumulation of undesirable uncleavable mutants. After library enrichment was observed by the on-yeast cleavage-capture assay, more precise selections were performed by fluorescence-activated cell sorting (FACS) to isolate pure yeast cells displaying interacting pairs.

Positive selection (MACS):

First, 10x diversity of affibody dimer displayed yeast cells were stained with Alexa Fluor 647-labeled anti-HA antibody (1:50 dilution in MACS buffer) at 4°C, and staining status was confirmed by flow-cytometry to ensure displayed cells are fully stained by antibody. After washing out residual antibodies, stained cells were incubated in 3C protease cleavage solution (0.4mg/mL 3C protease) at 4°C until the stained population reduced to 10% or less (the staining status can be monitored by flow-cytometry). The cleavage time is between 5 min to 30 min depending on how enriched the library is. Then, the cleaved cells were washed with MACS buffer, and 250 µL of Anti-Alexa Fluor 647 microbeads (Miltenyi, cat. no. 130-091-395) was added in cells with 10mL MACS buffer. After 10 min incubation, cells were passed through MACS LS columns (Miltenyi, cat. no. 130-042-401) attached to a magnetic stand (Miltenyi) and washed twice with 3mL PBE buffer (PBS, pH7.2, 0.5% bovine serum albumin, 2 mM EDTA). The elution was collected and grown in SDCAA overnight.

Negative selection (MACS):

10x diversity of affibody dimer displayed yeast cells were incubated in 3C protease cleavage solution for an hour at 4°C. After cleavage, cells were washed thoroughly with 10mL MACS buffer three times to remove cleaved affibodies from yeast library. 100 µL of biotinylated anti-HA Fab (Sigma, cat. no. 12158167001) was treated on yeast cells and incubated for an hour at 4°C, and staining status was confirmed by sampling small portion and mixing with Alexa Fluor 647 dye conjugated streptavidin (1:1000 dilution, lab-made) and running on flow cytometry. Next, the biotinylated anti-HA Fab stained cells were mixed with 250 µL streptavidin microbeads (Miltenyi, cat. no. 130-048-101) in 10mL MACS buffer for 30 min at 4°C. The yeast cells were then flowed over MACS LS columns (Miltenyi) and washed three times with 5mL PBE buffer. The flow-through was collected and grown in SDCAA overnight.

FACS sorting:

After 4 to 6 rounds of selection by MACS, FACS method was employed in subsequent rounds to further enrich the library. The staining and 3C protease cleavage methods remained the same as in the MACS selection process The cells were sorted by the FACS machine (Sony SH-800) to more accurately isolate the interacting pairs from the library. The population was divided into two groups based on antibody staining, with the antibody-stained group being sorted for positive selection and the unstained group being sorted for negative selection. Up to 5 $\times$ 10^6^ cells were sorted at a time during the positive selection by FACS to maintain the antibody staining level.

Deep sequencing of yeast libraries

DNA was isolated from 5-10 $\times$ 10^7^ yeast per round of selection by miniprep (Zymoprep II kit, Zymo Research). Individual 6-mer barcodes and random 8-mer sequences were added to the flanking regions of the sequencing product by PCR and amplified for 30 cycles. The amplicon region included library positions of both affibody A and B. An additional PCR amplification adding the Illumina primer sequences to produce final products containing Illumina P5-barcode-N8-read-Illumina P7. The amplified PCR product was purified by agarose gel purification and quantified by nanodrop, and deep sequenced by Illumina Miseq sequencer using a 2x300 V3 kit.

The amplicons were amplified using the following primers:

Illumina forward primer:

5'-AATGATACGGCGACCACCGAGATCTACACTCTTTCCCTACACGACGCTCTTCCGA-3'

Illumina reverse primer:

5'-CAAGCAGAAGACGGCATACGAGATCGGTCTCGGCATTCCTGCTGAACCGCTCTTC-3'

Sequence library filter

To identify the oligopeptide pairs with the highest likelihood of binding from the enriched library sequencing data, we used a one-sided hypergeometric test to filter the sequence libraries. This test checks for oligopeptide pairs that are sequenced more frequently than expected based on the abundance of the individual oligopeptide in the library. First, we defined the hypergeometric distribution (hygecdf(x,M,K,N); Matlab R2020a) with the following parameters, where *x* = number of times an oligopeptide pair is sequenced in the library; M = sum of all the number of times all oligopeptide pairs are sequenced in the library; K= number of times the first oligopeptide (i.e., attached to the yeast cell) is sequenced regardless of the second protein; N = number of times the second oligopeptide (i.e., HA-tagged) is sequenced regardless of the first protein. We then estimated a P-value using the survival function (1 - cumulative distribution function) for the specified distribution. The P value is defined as the cumulative probability (P(X > (n-1))) that the distribution takes a value greater than the number of times a protein pair is sequenced - 1. We used *p* < .05 as an unadjusted heuristic cutoff to keep enriched oligopeptide pairs and filter other potential non-specific pairs. After filtering, we generated a multiple sequence alignment (MSA) from the sequenced interface oligopeptide pairs for each library at each stage. The oligopeptide pairs were then pooled from multiple stages and counted once in the final MSA, regardless of the sequencing count, for subsequent coupling analyses.

X-ray crystallography

All protein complexes were reductively methylated (*51*), digested with carboxypeptidases A and B, purified by size exclusion chromatography, and concentrated. All well-diffracting crystals were grown by vapor diffusion with microseeding and were flash cooled in liquid nitrogen after cryoprotection. Initial, weakly diffracting crystals of LL1.c1 were formed in 2.5 M ammonium sulfate, 100 mM bis-tris-propane pH 7.0. These crystals were crushed and used to seed subsequent trials of this and other complexes, with crystals eventually grown using 150 nl protein (132 mg/ml), 120 nl of well solution (2.4 M ammonium sulfate, 100 mM bicine pH 9.0), and 30 nl seeds. Crystals were cryoprotected with 30% glycerol. Initial crystals of LL1.c4 were grown in 2.4 M ammonium phosphate dibasic, 100 mM Tris pH 8.5 and were used to seed the final crystal growth using 120 nl protein (167 mg/ml), 135 nl well solution (2.3 mM ammonium phosphate dibasic, 100 mM Tris pH 8.5), and 15 nl seeds. Crystals were cryoprotected with 30% glycerol. Crystals of LL1.c2 were grown with cross-seeding from LL1.c4 using 100 nl protein (120 mg/ml), 80 nl well solution (2.5 M ammonium sulfate, 100 mM sodium acetate pH 4.6), and 20 nl seeds. Crystals were cryoprotected with 3 M sodium malonate pH 5.0. Crystals of LL1.c6 were grown with cross-seeding from LL1.c1 using 120 nl protein (153 mg/ml), 105 nl well solution (2.5 M ammonium sulfate, 100 mM Tris pH 8.5), and 45 nl seeds. Crystals were cryoprotected with 3 M sodium malonate pH 8.5. Initial crystals of LL2.c7 were grown with cross-seeding from LL1.c1 using 100 nl protein (193 mg/ml), 80 nl well solution (2.9 M sodium malonate pH 5.0), and 20 nl seeds. These were used to microseed the final crystals, grown using 100 nl protein, 80 nl well solution (3 M ammonium sulfate, 100 mM HEPES pH 7.0). Crystals were cryoprotected with 3 M sodium malonate pH 7.0. Crystals of LL2.c1 were grown with cross-seeding from LL1.c7 using 105 nl protein (188 mg/ml), 135 nl well solution (2.5 M ammonium sulfate, 100 mM HEPES pH 7.0), and 15 nl seeds, which were then used to seed the final crystals from the same conditions. Crystals were cryoprotected with 30% glycerol. LL2.c3 was crystallized with cross-seeding from LL2.c7 using 100 nl protein (184 mg/ml), 80 nl well solution (2.4 M ammonium sulfate, 100 mM HEPES pH 7.0), and 20 nl seeds. Crystals were cryoprotected with 30% glycerol. LL2.c17 was crystallized with cross-seeding from LL1.c1 using 105 nl protein (132 mg/ml), 135 nl well solution (2.3 M ammonium phosphate dibasic, 100 mM Tris pH 8.2), and 15 nl seeds. Crystals were cryoprotected with 30% glycerol. Initial crystals of LL2.c22 were grown with cross-seeding from LL2.c7 using 100 nl protein (209 mg/ml), 80 nl well solution (3 M ammonium sulfate, 100 mM Tris pH 8.0), and 20 nl seeds. These were then used to seed the final crystals, grown using 105 nl protein, 120 nl well solution (2.5 M ammonium sulfate, 100 mM tris pH 8.2), and 30 nl seeds. Crystals were cryoprotected with 30% glycerol.

Diffraction data were collected at ALS beamline 5.0.2 (LL1.c1) and SSRL beamlines 12-1 (LL1.c2, LL1.c4, and LL1.c6) and 12-2 (LL2.1, LL2.c7, LL2.c17, and LL2.c22). Data were indexed, integrated, and scaled using either XDS (*52*) (LL1.c1, LL2.c1, LL2.c3, LL2.c7, LL2.c17, and LL2.c22) or Dials (*53*) (LL1.c2, LL1.c4, and LL1.c6). Spacegroups were assigned with pointless and reflections were merged with aimless from the CCP4 suite (*54*–*56*). All structures were solved by molecular replacement in Phaser (*57*) using the Zdk2 affibody (PDB id 5DJT) as a search model; translational noncrystallographic symmetry (tNCS) was detected for LL2.c1 and LL2.c7. Initial rebuilding was performed with phenix.autobuild (*58*) with subsequent cycles of interactive rebuilding in coot (*59*) and reciprocal space refinement in phenix (*60*–*62*) and Buster (*63*). For structures at <1.3 Å resolution, anisotropic B-factors were refined, while for structures at lower resolution, TLS groups were automatically assigned in phenix and refined. NCS restraints were used for LL1.c2 (*64*). tNCS correction in phenix was applied to LL2.c7. Final refinements of all structures were performed in phenix, and the resolution of the final refinement was chosen using the paired refinement technique as previously described (*65*). Model geometry was assessed with Molprobity (*66*). Atomic contacts were identified using a distance limit of 3.8 Å using contact from the CCP4 suite and by visual inspection. Packstat scores were calculated as the mean of 100 runs using Rosetta packstat (*67*). Crystallographic software used in this project was compiled and maintained by SBGrid (*68*). Crystallographic data collection and refinement statistics along with PDB deposition codes are reported in tables S1 and S2.

Visualization

PyMOL

GraphPad Prism 9

BioRender (https://biorender.com/)

WebLogo 3 (http://weblogo.threeplusone.com/)

Igraph 0.10.2

Circlize 0.4.15

Origin 7.0


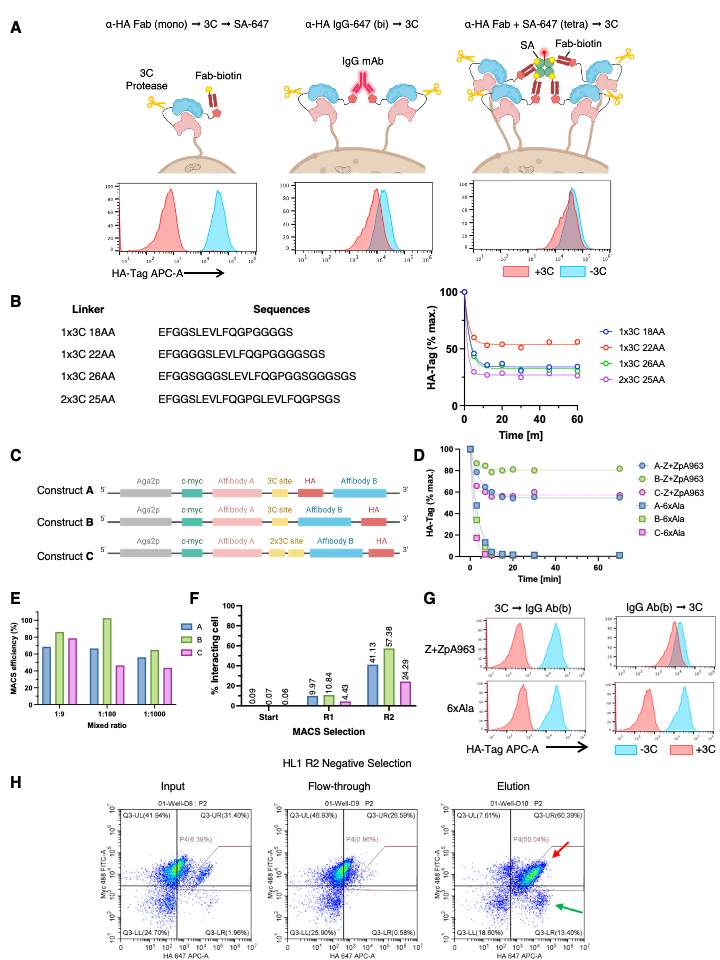
 **Fig. S1. Design and optimization of synthetic coevolution strategy**

(A) Detailed schematics of the cleavage-capture assay. Note that the dimer complex can be stabilized by avidity effects of polyvalent antibodies binding to the C-terminal.

(B) Multiple linker lengths (18-26AA) in combination with one or two 3C protease sites were tested by on-yeast cleavage-capture assay. 22AA linker with 1 copy of 3C protease site showed the most stable HA-tag antibody staining level during 3C protease cleavage.

(C) Construct design of three different constructs of Z+ZpA963 pair.

(D) On-yeast cleavage-capture assay of three constructs with interacting (Z+ZpA963) and non-interacting (6xAla) pairs.

(E) Interacting pair recovery efficiency during MACS with a mock library pool. Interacting pair and non-interacting pair cells are mixed at different ratios (1:9, 1:100, and 1:1000).

(F) Interacting pair enrichment during two rounds of MACS with mock library which is mixed at 1:1000 ratio. The frequencies of interacting pairs at each round were plotted.

(G) When 3C protease was added to dimer-displaying cells before HA-tag antibody staining, both interacting pair (Z+ZpA963) and non-interacting pair (6xAla) displaying cells were not able to be stained with anti-HA tag antibody (left), whereas if the antibody was added to cells before 3C protease cleavage, only interacting pair-displaying cells retain HA-tag antibody staining (right).

(H) Negative selection. Flow cytometry dot plots showing how negative selection can remove mutants from the library. If yeast cells displaying the protein dimer library were treated with 3C protease first and vigorously washed multiple times, uncleavable mutants including 3C site mutations or deletions could be labeled by anti-HA antibody and subsequently removed by MACS. After negative selection, each fraction of cells was grown in SD-CAA media, and protein display was re-induced with SG-CAA media growth. After 24 hrs post-induction, cells were stained with anti-Myc tag antibody and anti-HA tag antibody, then cleaved with 3C protease to monitor their staining level change. Mutants with 3C mutation (red arrow) and deletion (green arrow) were efficiently separated in the elution fraction and confirmed by DNA sequencing.

**
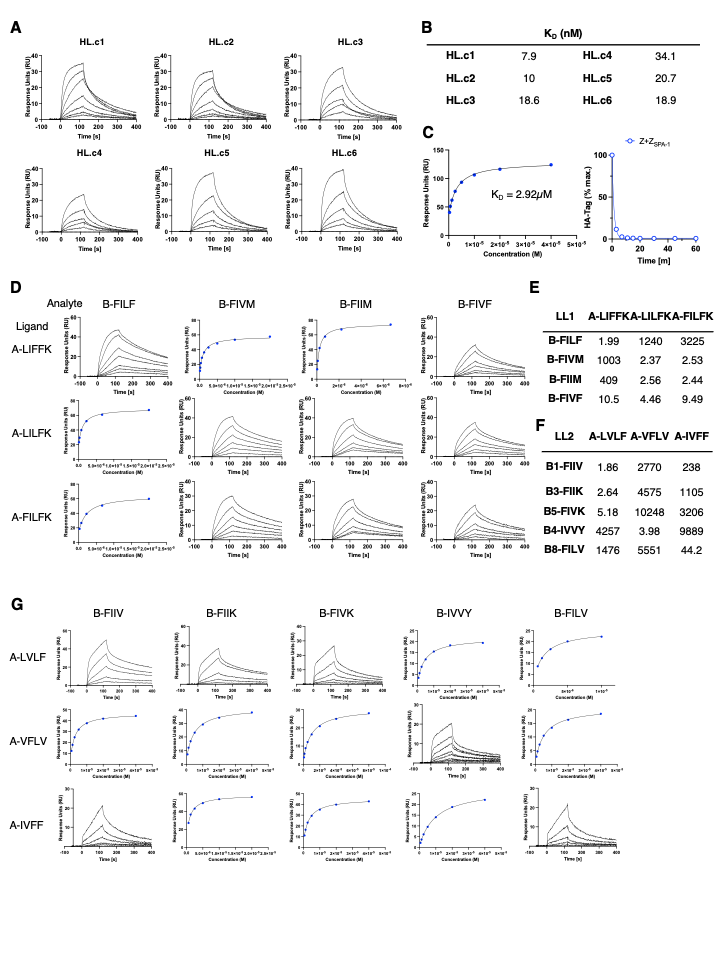
**

**Fig. S2. Binding affinity of coevolved variants**

(A-B) The kinetic binding curves of coevolved pairs from HL1 and HL2 libraries, as measured by SPR, and K_D_ value. We measured binding affinities using surface plasmon resonance (SPR) that ranged from 7.9 nM to 34.1 nM, similar to the original template dimer affinity of 10 nM.

(C) The binding affinity (left) and on-yeast cleavage-capture assay (right) of Z and Z_SPA-1_. The Z+ Z_SPA-1_ pair rapidly lost its HA-tag fluorescence within 15 min of 3C protease treatment (right).

(D-E) The kinetic binding curves (range from 25 nM to 0.3906 nM) and fitted curves of coevolved pairs from LL1 library, as measured by SPR and K_D_ value.

(F-G) The kinetic binding curves (range from 27.4 nM to 0.339 nM) and fitted curves of coevolved pairs from LL2 library, as measured by SPR and K_D_ value. The K_D_ values were determined by steady-state model or 1:1 binding model using the Biacore evaluation software.

**
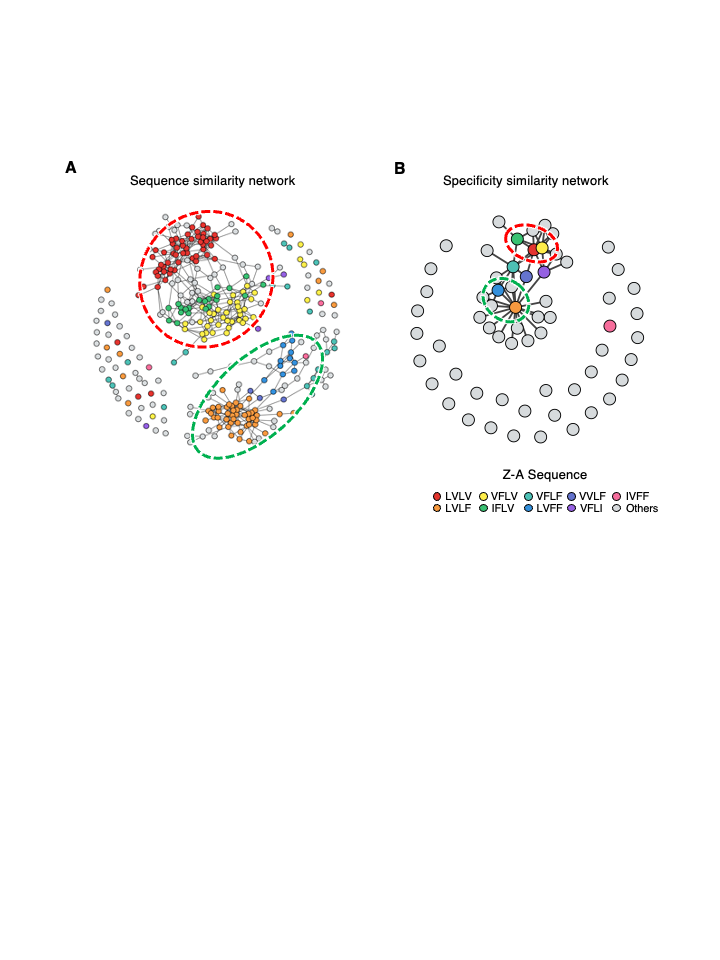
Fig. S3 Correlation between sequence similarity network and specificity similarity network**

(A) Sequence similarity networks of concatenated 8 amino acid Z-A/Z-B library position sequences from LL2 round NGS data. The edit distance threshold for connecting nodes in the network is 1.

(B) Specificity similarity network of Z-A mutants from LL2 round 6 and 7 sequence data. Two Z-A sequences are connected if they share at least two Z-B binding partners in common.

The color codes of notable Z-A sequences for both plots are provided at the bottom. Red dashed lines from the both networks indicate close connectivity between three Z-A sequences (LVLV, VFLV, IFLV), and green dashed lines indicate another group of Z-A sequences (LVLF and LVFF). This comparison supports the separation between two groups in sequence similarity also correlates with distance from specificity similarity network.

Red dashed circles in both networks indicate a group of three Z-A sequences (LVLV, VFLV, IFLV), and green dashed circles indicate another separated group of Z-A sequences (LVLF and LVFF). This comparison supports the observation that the sequence similarity network can provide insights into the binding specificity of Z-A sequences.


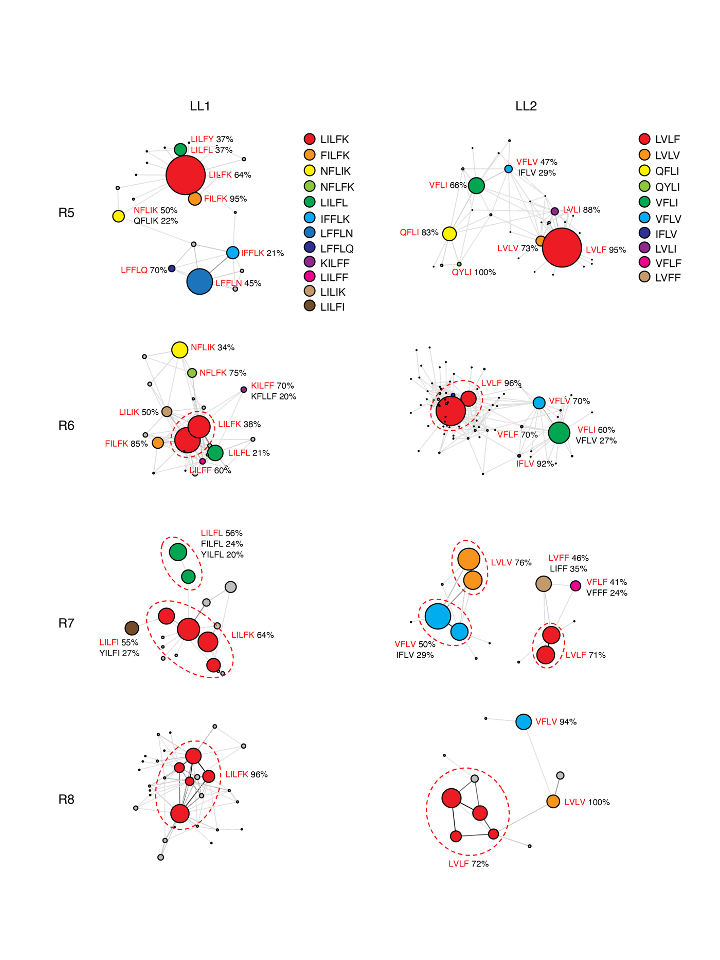
 **Fig. S4. Visualization of NGS data: cluster graphs**

Each clustered community in the Z-A+Z-B (9 amino acids for LL1 and 8 amino acids for LL2) sequence similarity network is contracted to a single node to depict cluster graphs of the late four screening rounds (R5-R8). The edit distance threshold (Dt) to form edges is 2 for R5 and R6 (Dt $\leq$ 2), and 1 for R7 and R8 (Dt $\leq$ 1). The nodes are colored based on the dominant Z-A sequences of each node. The isolated nodes were not included in the figure. The cluster graphs, which contract each clustered community into a single node, can efficiently show such relationships between co-evolved mutants and the structure of coevolutionary networks throughout the different screening rounds.

**
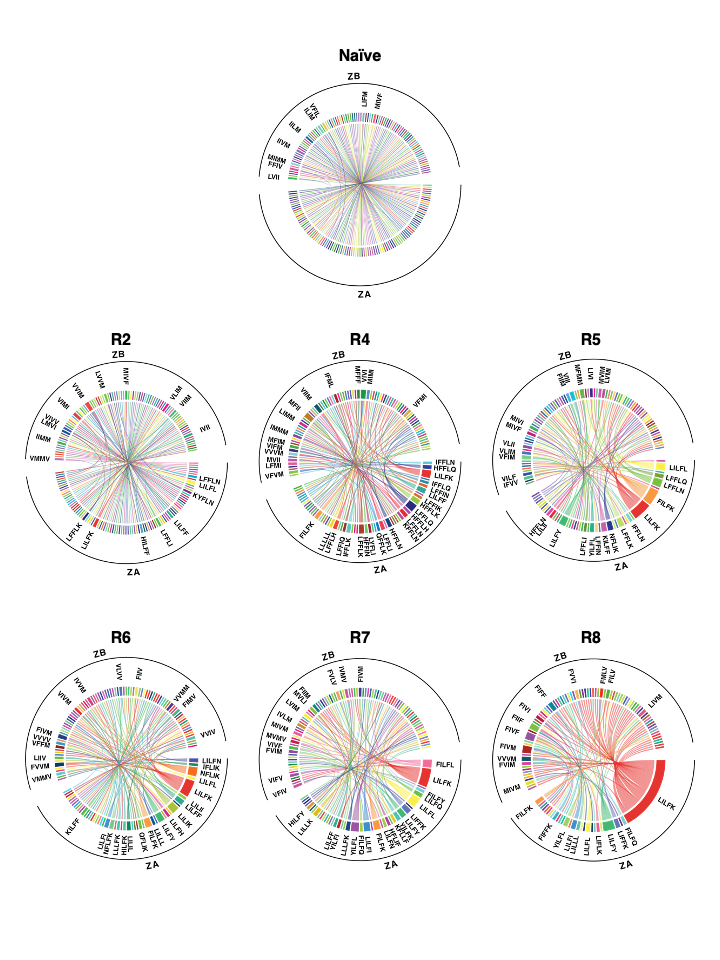
 Fig. S5. Visualization of NGS data: cross-reactivity Circos plots of LL1 library**

The Circos plots of all LL1 screening rounds illustrate the progressive change of pairwise relationships between the 100 sampled pairs of Z-A and Z-B proteins. Each pair is normalized to have equal area, providing a visual representation of the approximate cross-reactivity of each sequence.

**
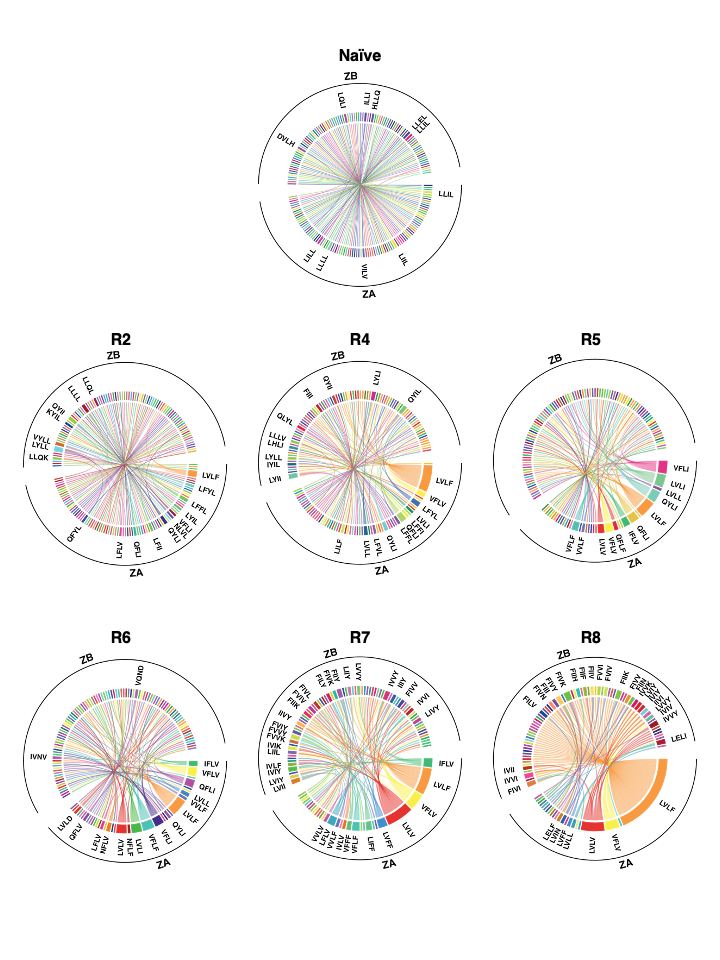
**

**Fig. S6. Visualization of NGS data: cross-reactivity Circos plots of LL2 library**

The Circos plots of all LL2 screening rounds illustrate the progressive change of pairwise relationships between the 100 sampled pairs of Z-A and Z-B proteins. Each pair is normalized to have equal area, providing a visual representation of the approximate cross-reactivity of each sequence.


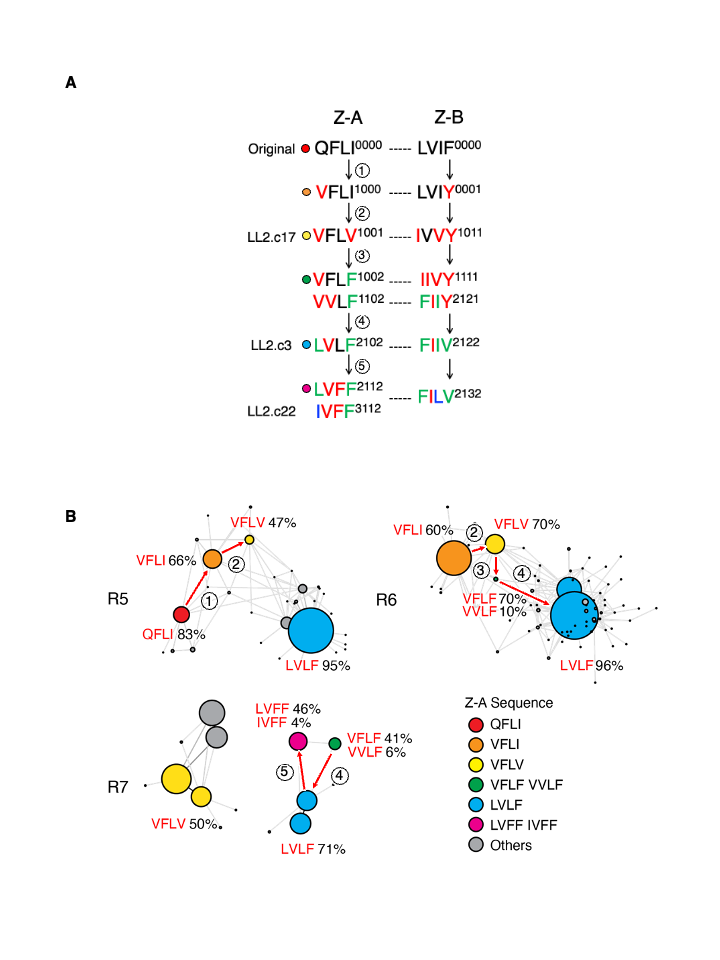


**Fig. S7. Visualization of LL2 mutational pathway on SSN**

The connectivity between Z-A mutants from the mutational pathway shown in (A) were visualized from LL2 R5-R7 SSN cluster graphs (B). Color coding of each mutant is the same as noted in (B), and each mutagenesis step (steps 1 to 5, as noted in circled numbers) is designated by red arrows. Node color represents the dominant Z-A sequence of the clustered community, and grey communities have different dominant sequences outside the Z-A mutational pathway. The percentage of the designated Z-A dominant sequence in each community is labeled next to each node. The node size reflects the relative number of sequences in each clustered community.


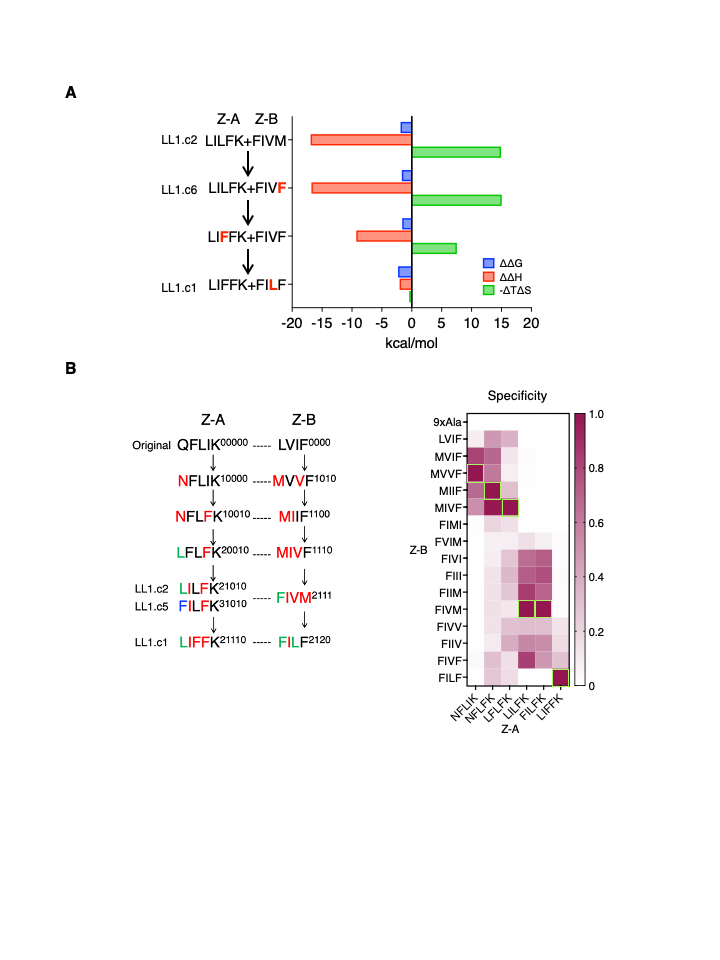


**Fig. S8. Thermodynamic analysis and specificity changes of mutants from the LL1 mutational pathway**

(A) A plot illustrating the changes in ∆∆G, ∆∆H, and -∆T∆S for four LL1 mutants compared to the original pair (QFLIK/LVIF). Mutations introduced in each step are highlighted in red.

(B) A single mutational pathway (left) of mutants from the LL1 library connecting the original sequence (QFLIK/LVIF) with the prominent LL1 library mutants. Mutated positions are color-coded: red (one mutation), green (two mutations), and blue (three mutations). The number of mutations at each position is represented by a 4-digit number next to each Z-A and Z-B sequence. A matrix (right) to show binding specificity changes of the Z-A variants from the pathway. Binding affinities measured by on-yeast cleavage-capture assay were normalized based on the highest affinity in each Z-A sequence. The highest affinity in each column was boxed in green. Control is a mutant that all library positions are mutated to alanines. Data are mean of n = 3 independent replicates.


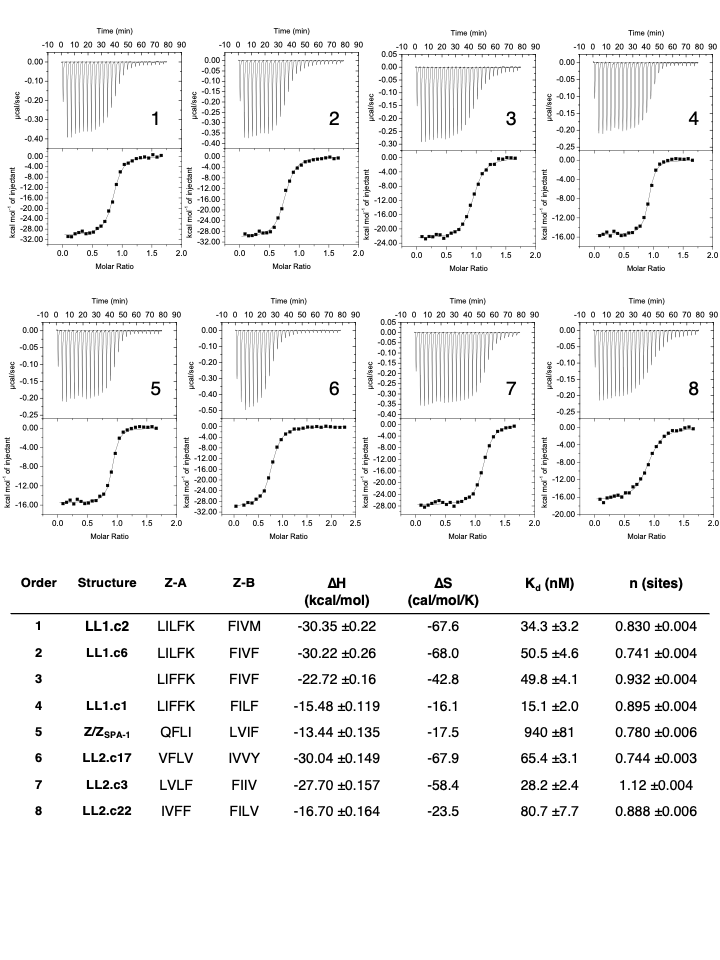


**Fig. S9. Isothermal Titration Calorimetry.**

Plots of enthalpy change and binding isotherms for Z domain-affibody pairs. For each titration, Z-B chains in the calorimeter cell were titrated by injecting Z-A chains at 7 to 10-fold higher concentration at 298 K. Heats of dilution of Z-A chains injected into buffer were subtracted before fitting binding isotherms.

**
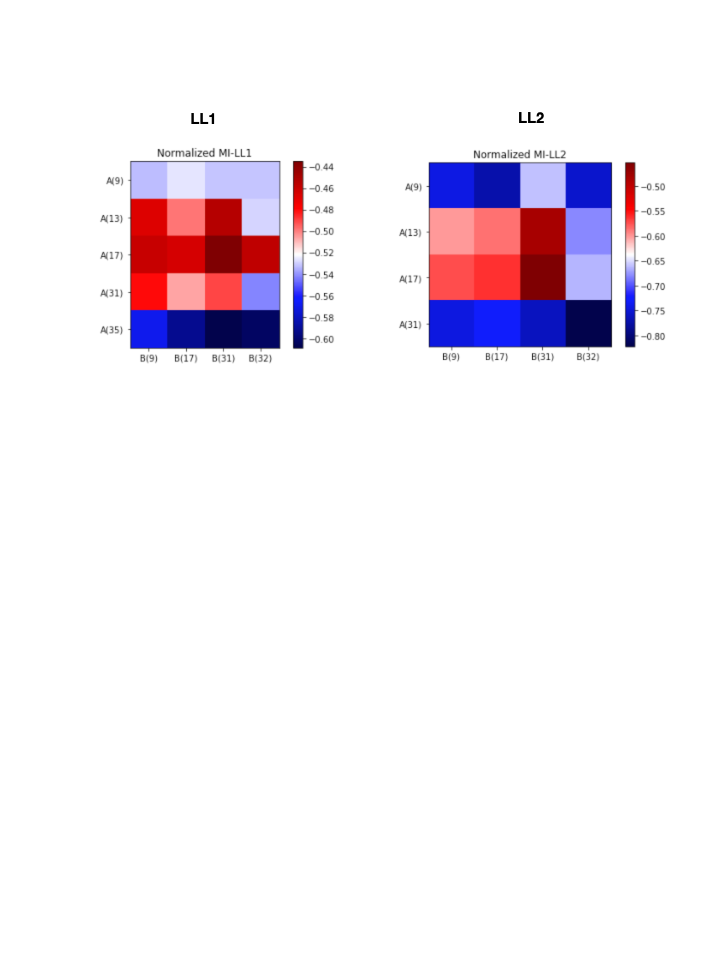
**

**Fig. S10. Mutual information matrix**

(A) Normalized mutual information matrix of p-value < 0.05 filtered round 6 and round 7 sequencing data.


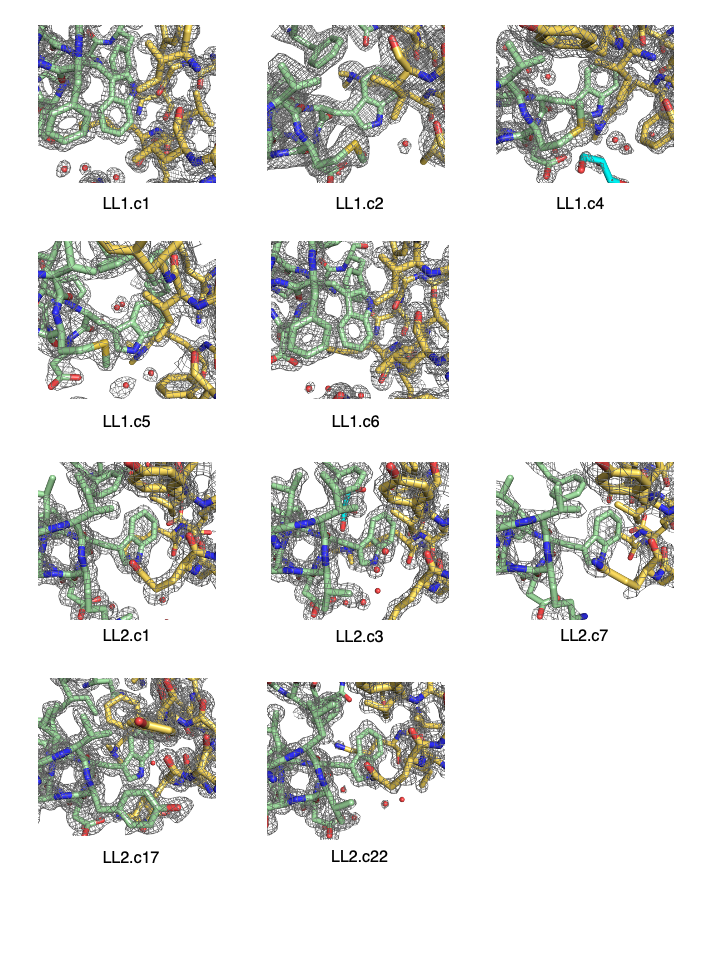
 **Fig. S11. Representative electron density maps at the Z-A/Z-B interface**

2mFo – DFc maps (gray) are contoured at 1.5 σ over Z-B (pale green), Z-A(yellow-orange), ordered waters (red balls), and other solvent molecules (cyan). Some atoms are omitted for clarity.


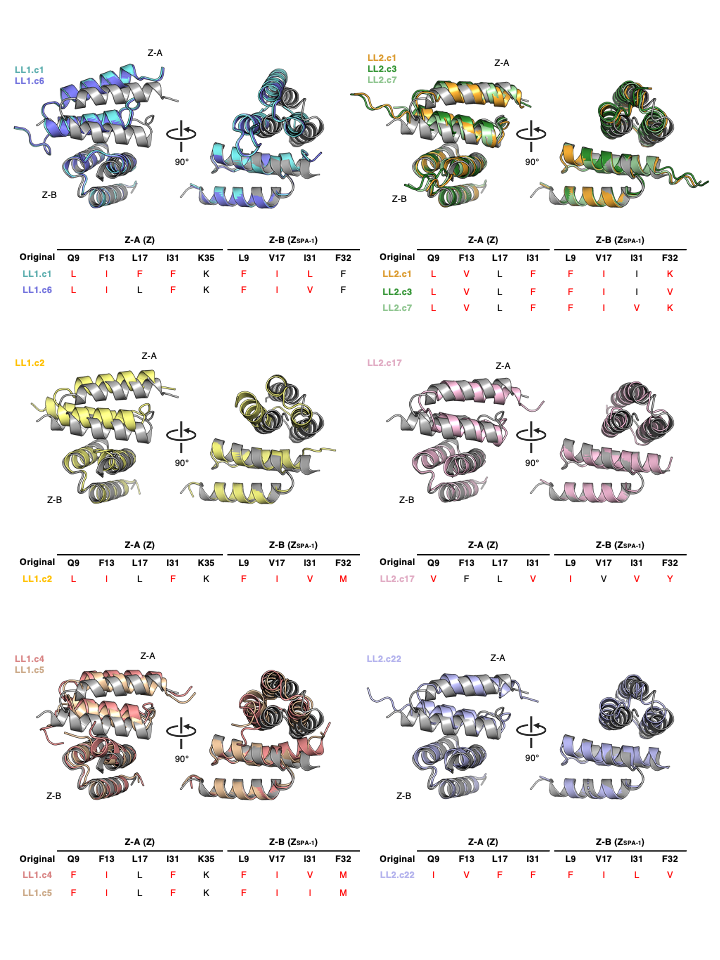
 **Fig. S12. Structural comparison of coevolved variants and original dimer, Z+Z_SPA-1_** Superposition of original dimer (grey) with each mutant from LL1 and LL2 libraries. Color coding of the structures is denoted in the top left of each panel. The amino acid sequences in library positions are represented below each structure.


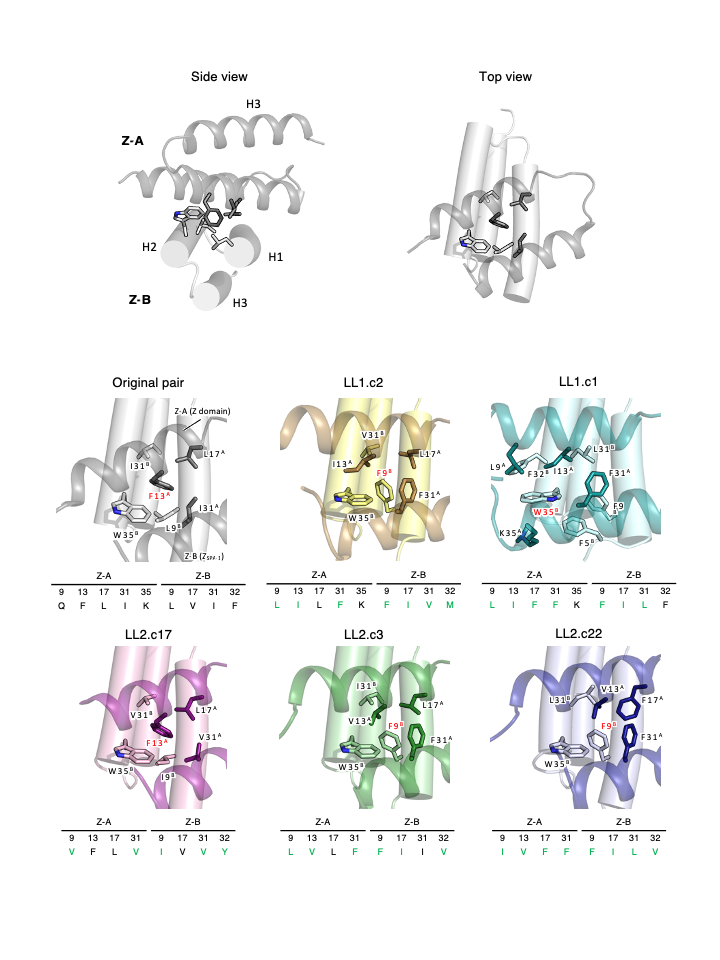
 **Fig. S13. Structural comparison of the central hydrophobic patch of coevolved variants and original dimer, Z+Z_SPA-1_.** Close-up views of the main hydrophobic patch of the original dimer and mutants. The interfaces were viewed from the top to fully depict all residues at once with helix 3 of the Z-A chains omitted for clarity. Each residue is labeled with its associated monomer, A or B, in superscript. The core residue of each hydrophobic patch is labeled in red. Z-B helices are represented by cartoon cylinders.


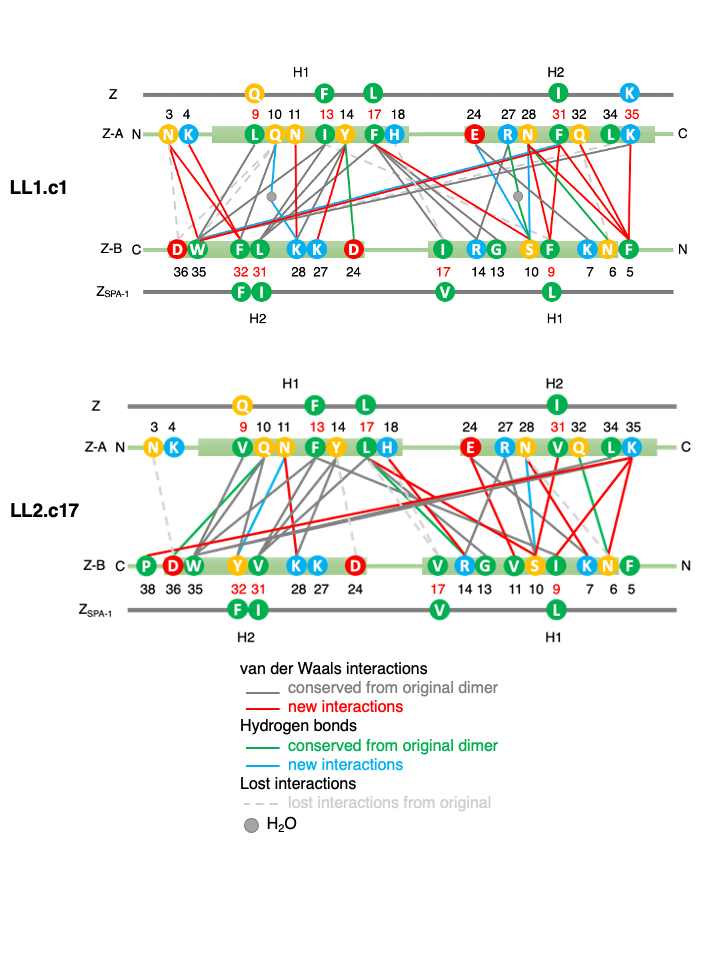
 **Fig. S14. Diagram of contacts between Z-A and Z-B domains of LL1.c1 (LIFFK/FILF) and LL2.c17 (VFLV/IVVY) in comparison with the original pair, Z/Z_SPA-1_.** Position numbers colored in red are library positions. Line color represents the properties of each interaction and conservation or change of interactions compared to the original dimer. Amino acid residues depicted in color are at the interface between A and B domains (within 4.0 Å), with colors reflecting the properties of the side chain: positively charged (blue), negatively charged (red), hydrophobic (green), and polar (yellow).

Table S1. Crystallographic Data collection and refinement statistics for LL1 structures.

|  | **LL1.c1** | **LL1.c2** | **LL1.c4** | **LL1.c5** | **LL1.c6** |
| --- | --- | --- | --- | --- | --- |
| **PDB ID** | 8DA3 | 8DA4 | 8DA5 | 8DA6 | 8DA7 |
| **Wavelength (Å)** | 1 | 0.7749 | 0.7749 | 0.7749 | 0.7749 |
| **Resolution range** | 36.86 - 1.06 (1.098 - 1.06) | 45.37 - 1.92 (1.989 - 1.92) | 40.03 - 1.0 (1.036 - 1.0) | 35.8 - 1.5 (1.554 - 1.5) | 27.26 - 1.02 (1.056 - 1.02) |
| **Space group** | P 21 21 21 | P 41 21 2 | P 21 21 21 | P 21 21 21 | P 21 21 21 |
| **Unit cell (a,b,c (Å)) (α,β,γ (°))** | 35.5456 42.449 74.2884 90 90 90 | 72.449 72.449 181.469 90 90 90 | 38.6123 41.1531 160.108 90 90 90 | 37.016 42.105 140.805 90 90 90 | 35.608 42.366 74.202 90 90 90 |
| **Total reflections** | 571621 (24576) | 935627 (64228) | 1756952 (171607) | 434578 (43548) | 465164 (46776) |
| **Unique reflections** | 51100 (4466) | 37848 (3691) | 138513 (13660) | 36176 (3545) | 57264 (5584) |
| **Multiplicity** | 11.2 (5.5) | 24.7 (17.4) | 12.7 (12.6) | 12.0 (12.3) | 8.1 (8.4) |
| **Completeness (%)** | 98.53 (87.01) | 99.72 (99.41) | 99.11 (98.87) | 99.85 (99.66) | 98.75 (97.38) |
| **Mean I/sigma(I)** | 13.77 (1.14) | 15.82 (0.91) | 10.75 (0.70) | 20.23 (0.95) | 12.91 (0.80) |
| **Wilson B-factor** | 10.89 | 42.60 | 13.10 | 28.59 | 13.12 |
| **R-merge** | 0.07684 (1.562) | 0.1035 (3.227) | 0.07674 (2.947) | 0.06605 (3.09) | 0.06462 (2.47) |
| **R-meas** | 0.08051 (1.73) | 0.1056 (3.324) | 0.07997 (3.073) | 0.06898 (3.223) | 0.06915 (2.631) |
| **R-pim** | 0.02358 (0.7217) | 0.02097 (0.7771) | 0.02219 (0.8613) | 0.01961 (0.9039) | 0.02412 (0.8953) |
| **CC1/2** | 0.999 (0.514) | 1 (0.464) | 0.999 (0.657) | 0.999 (0.452) | 0.999 (0.456) |
| **CC*** | 1 (0.824) | 1 (0.796) | 1 (0.891) | 1 (0.789) | 1 (0.791) |
| **Reflections used in refinement** | 51040 (4427) | 37765 (3676) | 137574 (13540) | 36170 (3541) | 57228 (5571) |
| **Reflections used for R-free** | 2574 (225) | 1856 (172) | 6857 (674) | 1996 (196) | 2873 (251) |
| **R-work** | 0.1469 (0.2860) | 0.2067 (0.3436) | 0.1681 (0.3935) | 0.2160 (0.3901) | 0.1617 (0.3525) |
| **R-free** | 0.1675 (0.3202) | 0.2471 (0.3996) | 0.1915 (0.4167) | 0.2507 (0.4148) | 0.1844 (0.4045) |
| **Number of non-hydrogen atoms** | 1161 | 2742 | 2339 | 1915 | 1123 |
| **macromolecules** | 981 | 2629 | 1948 | 1803 | 947 |
| **ligands** | 23 | 5 | 14 | 0 | 9 |
| **solvent** | 161 | 108 | 385 | 112 | 169 |
| **Protein residues** | 113 | 330 | 236 | 227 | 114 |
| **RMS(bonds)** | 0.009 | 0.015 | 0.016 | 0.008 | 0.016 |
| **RMS(angles)** | 1.19 | 1.37 | 1.42 | 0.93 | 1.47 |
| **Ramachandran favored (%)** | 98.86 | 98.95 | 100.00 | 100.00 | 98.91 |
| **Ramachandran allowed (%)** | 1.14 | 1.05 | 0.00 | 0.00 | 1.09 |
| **Ramachandran outliers (%)** | 0.00 | 0.00 | 0.00 | 0.00 | 0.00 |
| **Rotamer outliers (%)** | 1.03 | 1.13 | 0.00 | 1.09 | 0.00 |
| **Clashscore** | 1.99 | 3.08 | 0.51 | 1.97 | 1.57 |
| **Average B-factor** | 17.83 | 57.30 | 19.70 | 39.93 | 20.71 |
| **macromolecules** | 15.17 | 57.30 | 17.50 | 39.67 | 18.51 |
| **ligands** | 65.86 | 143.99 | 46.60 |  | 48.37 |
| **solvent** | 28.39 | 53.16 | 30.43 | 44.17 | 31.92 |
| **Number of TLS groups** | - | 16 | - | 11 | - |

Statistics for the highest-resolution shell are shown in parentheses.

Table S2. Crystallographic Data collection and refinement statistics for LL2 structures.

|  | LL2.c1 | LL2.c3 | LL2.c7 | LL2.c17 | LL2.22 |
| --- | --- | --- | --- | --- | --- |
| **PDB ID** | 8DA8 | 8DA9 | 8DAA | 8DAB | 8DAC |
| **Wavelength** | 0.8265 | 0.8856 | 0.9795 | 0.7749 | 0.8856 |
| **Resolution range** | 26.46 - 1.29 (1.336 - 1.29) | 34.2 - 1.35 (1.398 - 1.35) | 22.64 - 1.75 (1.813 - 1.75) | 31.65 - 1.134 (1.175 - 1.134)  (1.254 - 1.134)^a^ | 36.93 - 1.19 (1.233 - 1.19) |
| **Ellipsoidal^a^ resolution limit (Å) (direction)^b^** | - | - | - | 1.133 (0.834 a* - 0.552 c*)  1.594 (b*)  1.205 (0.860 a* + 0.511 c*) | - |
| **Space group** | C 1 2 1 | C 1 2 1 | C 1 2 1 | C 1 2 1 | C 1 2 1 |
| **Unit cell (a,b,c (Å)) (α,β,γ (°))** | 50.251 54.556 34.721 90 97.638 90 | 53.403 54.259 68.601 90 94.414 90 | 49.588 53.023 68.662 90 98.388 90 | 77.091 35.532 49.433 90 115.421 90 | 50.443 54.676 34.637 90 96.902 90 |
| **Total reflections** | 157165 (16068) | 279261 (27130) | 64587 (6488) | 423725 (39834) | 184914 (10853) |
| **Unique reflections** | 22160 (2223) | 41323 (4054) | 17068 (1711) | 43360 (140) | 28352 (2038) |
| **Multiplicity** | 7.1 (7.2) | 6.8 (6.7) | 3.8 (3.8) | 9.8 (9.4) | 6.5 (5.3) |
| **Completeness (%)** | 94.39 (94.25) | 95.84 (93.56) | 95.16 (95.52) | 60.77 (3.10) | 94.27 (66.45) |
| **Completeness (ellipsoidal)^c^ (%)** | - | - | - | 89.54 (56.95) | - |
| **Mean I/sigma(I)** | 17.31 (1.86) | 8.93 (0.76) | 6.49 (0.61) | 5.12 (0.07) | 12.62 (0.66) |
| **Mean I/sigma(I) (ellipsoidal)^a^** | - | - | - | 15.86 (1.21) | - |
| **Wilson B-factor** | 16.05 | 17.62 | 30.47 | 12.19 | 14.64 |
| **R-merge** | 0.06021 (1.304) | 0.1129 (3.679) | 0.2109 (1.973) | 0.09152 (4.141) | 0.06903 (2.251) |
| **R-meas** | 0.06502 (1.404) | 0.1224 (3.989) | 0.2443 (2.297) | 0.09654 (4.383) | 0.07499 (2.494) |
| **R-pim** | 0.02429 (0.5168) | 0.0467 (1.522) | 0.1212 (1.158) | 0.03031 (1.415) | 0.0289 (1.053) |
| **CC1/2** | 0.999 (0.824) | 0.997 (0.172) | 0.991 (0.356) | 0.999 (0.321) | 0.999 (0.365) |
| **CC*** | 1 (0.951) | 0.999 (0.542) | 0.998 (0.725) | 1 (0.697) | 1 (0.731) |
| **Reflections used in refinement** | 22116 (2213) | 41189 (4007) | 17025 (1706) | 27307 (139) | 28243 (1981) |
| **Reflections used for R-free** | 1128 (115) | 1640 (161) | 1704 (171) | 1528 (8) | 1433 (96) |
| **R-work** | 0.2136 (0.3587) | 0.1756 (0.3640) | 0.2509 (0.4241) | 0.1717 (0.3678) | 0.1605 (0.4454) |
| **R-free** | 0.2351 (0.4007) | 0.1982 (0.3978) | 0.2980 (0.4255) | 0.1993 (0.4122) | 0.1970 (0.5407) |
| **Number of non-hydrogen atoms** | 1021 | 1998 | 1752 | 1033 | 1037 |
| **macromolecules** | 918 | 1837 | 1712 | 885 | 920 |
| **ligands** | 14 | 41 | 7 | 0 | 13 |
| **solvent** | 97 | 134 | 33 | 148 | 111 |
| **Protein residues** | 114 | 232 | 227 | 107 | 116 |
| **RMS(bonds)** | 0.012 | 0.012 | 0.002 | 0.007 | 0.019 |
| **RMS(angles)** | 0.014 | 0.014 | 0.47 | 0.83 | 1.46 |
| **Ramachandran favored (%)** | 100.00 | 100.00 | 99.53 | 100.00 | 100.00 |
| **Ramachandran allowed (%)** | 0.00 | 0.00 | 0.47 | 0.00 | 0.00 |
| **Ramachandran outliers (%)** | 0.00 | 0.00 | 0.00 | 0.00 | 0.00 |
| **Rotamer outliers (%)** | 3.16 | 1.08 | 2.92 | 2.35 | 0.00 |
| **Clashscore** | 2.18 | 1.08 | 3.30 | 1.13 | 4.41 |
| **Average B-factor** | 22.60 | 23.47 | 36.00 | 18.88 | 20.28 |
| **macromolecules** | 21.73 | 22.49 | 35.93 | 17.24 | 18.77 |
| **ligands** | 27.79 | 48.69 | 54.69 |  | 36.65 |
| **solvent** | 30.53 | 31.85 | 35.36 | 28.68 | 31.92 |
| **Number of TLS groups** | - | 14 | 13 | - | - |

Statistics for the highest-resolution shell are shown in parentheses.

^a^ These statistics are for data that were truncated by STARANISO to remove poorly measured reflections affected by anisotropy.

^b^ The resolution limits for three directions in reciprocal space are indicated here. To accomplish this, STARANISO computed an ellipsoid postfitted by least squares to the cutoff surface, removing points where the fit was poor. Note that the cutoff surface is unlikely to be perfectly ellipsoidal, so this is only an estimate.

^c^ The anisotropic completeness was obtained by least squares fitting an ellipsoid to the reciprocal lattice points at the cutoff surface defined by a local mean *I*/σ*I* threshold of 1.0, rejecting outliers in the fit due to spurious deviations (including any cusp), and calculating the fraction of observed data lying inside the ellipsoid so defined. Note that the cutoff surface is unlikely to be perfectly ellipsoidal, so this is only an estimate.

| **Complex** | **Z-A** | **Z-B** | **PackStat** | **Cα r.m.s.d^c^ (Å)** | **BSA (Å^2^)** | **Polar BSA (Å^2^)** | **Nonpolar BSA (Å^2^)** | **vDW contacts** | **Hydrogen bonds** |
| --- | --- | --- | --- | --- | --- | --- | --- | --- | --- |
| **Z/Z_SPA-1_** | QFLI | LVIF | 0.640 | - | 1678 | 695 | 983 | 60 | 8 |
| **LL1.c1** | LIFFK | FILF | 0.685 | 1.04 | 1264 | 401 | 863 | 65 | 3 |
| **LL1.c2^a^** | LILFK | FIVM | 0.694 | 1.382 | 1163 | 373 | 790 | 49 | 5 |
| **LL1.c4^b^** | FILFK | FIVM | 0.692 | 1.343 | 1395 | 469.5 | 925.5 | 80 | 9 |
| **LL1.c5^b^** | FILFK | FIVIM | 0.718 | 1.597 | 1470.5 | 502.5 | 968 | 61 | 5 |
| **LL1.c6** | LILFK | FIVF | 0.671 | 1.05 | 1474 | 510 | 964 | 43 | 5 |
| **LL2.c1** | LVLF | FIIK | 0.681 | 0.659 | 1257 | 524 | 733 | 40 | 3 |
| **LL2.c3^b^** | LVLF | FIIV | 0.671 | 0.642 | 1167 | 405.5 | 761.5 | 40 | 3 |
| **LL2.c7^b^** | LVLF | FIVK | 0.638 | 0.622 | 1345.5 | 500.5 | 845 | 41 | 2 |
| **LL2.c17** | VFLV | IVVY | 0.775 | 0.593 | 1493 | 571 | 922 | 66 | 7 |
| **LL2.c22** | IVFF | FILV | 0.676 | 0.625 | 1282 | 477 | 805 | 33 | 5 |

**Table S3. Atomic contacts and dimer geometry changes (Cα r.m.s.d.) of mutant crystal structures compared to parent structure (Z+Z_SPA-1_).**

^a^ mean of three complexes in the asymmetric unit

^b^ mean of two complexes in the asymmetric unit

^c^ When affibody domains are aligned, Cα r.m.s.d. of Z domain compared to the parent structure (PDB: 1LP1) was calculated to measure the relative change of packing geometry.
